# Supplementary material for: Predicting areas important for ecological connectivity throughout Canada
Source: PLoS One. 2023 Feb 22;18(2):e0281980. doi: 10.1371/journal.pone.0281980 (PMC9946242; doi:10.1371/journal.pone.0281980)
Supplement: S1 Table — Movement costs were assigned to anthropogenic layers using our own knowledge but in consultation with members of the Canadian Connectivity Working Group (https://www.conservation2020canada.ca/connectivity). Costs for natural features were assigned based on published data, indicated in the last column, and in consultation with experts (Jodi Hilty, Clayton Lamb). CHF–Canadian Human Footprint, GHF = Global Human Footprint. (DOCX) [file pone.0281980.s001.docx]

| **LAYER** | **COUNTRY** | **COST** | **LAYER SOURCE** | **REFERENCES FOR COSTS** |
| --- | --- | --- | --- | --- |
| CHF - Built environments | Canada | 1000 | Agriculture and Agri-Food Canada; Science and Technology Branch 2016 |  |
| CHF - Croplands | Canada | 100 | Agriculture and Agri-Food Canada; Science and Technology Branch 2016 |  |
| CHF - Dams | Canada | 1000 | Global Forest Watch Canada 2010 |  |
| CHF - Forestry (cut between 1985 & 2015) | Canada | 10 | White, J. C., Wulder, M. A., Hermosilla, T., Coops, N. C. & Hobart, G. W. A nationwide annual characterization of 25years of forest disturbance and recovery for Canada using Landsat time series. Remote Sensing of Environment 194, 303–321 (2017). |  |
| CHF - Mining | Canada | 1000 | Government of Canada; Natural Resources Canada 2017 |  |
| CHF - Nighttime lights | Canada | 1000 | Annual composite from 2016 generated by NOAA to assess nighttime lights |  |
| CHF - Oil and gas | Canada | 1000 | Natural Resources Canada 2017 |  |
| CHF - Pasturelands | Canada | 10 | Agriculture and Agri-Food Canada; Science and Technology Branch 2016 |  |
| Lakes >= 10ha | Canada | 1000 | Lehner, B., and M. L. Messager. 2016. HydroLAKES Technical Documentation Version 1.0. https://www.hydrosheds.org/page/hydrolakes | 6, 34, 48 |
| Rails | Canada | 1000 | Natural Resources Canada’s National Railway Network (2012) |  |
| Roads - minor | Canada | 10 | Poley, L., Schuster, R., Smith, R. & Ray, J. Identifying Differences in Roadless Areas in Canada Based on Global, National, and Regional Road Datasets. In review. |  |
| Roads - two-lane highway | Canada | 100 |  |  |
| Roads - multi-lane highways | Canada | 1000 |  |  |
| Elevation > 2300m | Canada & U.S. | 1000 | Global Multi-resolution Terrain Elevation Dataset, USGS 2010 | 40, 41, 45 |
| Glaciers | Canada & U.S. | 1000 | CanVec Series, Hydrographic Features 2017 |  |
| Ocean | Canada & U.S. | 1000 | NRCan Atlas of Canada Data (2017) |  |
| Rivers > 28m3/sec | Canada & U.S. | 1000 | HydroRIVERS : Lehner, B. & Grill, G. Global river hydrography and network routing: baseline data and new approaches to study the world’s large river systems. Hydrological Processes 27, 2171–2186 (2013). | 6, 34, 48 |
| Sea Ice | Canada & U.S. | 10 | USGS North America Glaciers and Sea Ice | 49 |
| Slopes > 30 degrees | Canada & U.S. | 1000 | Global Multi-resolution Terrain Elevation Dataset, USGS 2010 | 39, 42, 45-47 |
| GHF - Built environments | U.S. | 1000 | Venter, O. et al. Sixteen years of change in the global terrestrial human footprint and implications for biodiversity conservation. Nat Commun 7, 12558 (2016). |  |
| GHF - Croplands | U.S. | 100 |  |  |
| GHF - Nightime lights | U.S. | 1000 |  |  |
| GHF - Pasturelands | U.S. | 10 |  |  |
| GHF - Rails | U.S. | 1000 |  |  |
| GHF - Road buffer (500 - 1000m) | U.S. | 1 |  |  |
| GHF - Road buffer (500m) | U.S. | 100 |  |  |
| GHF - Roads | U.S. | 1000 |  |  |
