# Supplementary material for: Predicting areas important for ecological connectivity throughout Canada
Source: PLoS One. 2023 Feb 22;18(2):e0281980. doi: 10.1371/journal.pone.0281980 (PMC9946242; doi:10.1371/journal.pone.0281980)
Supplement: S2 Table — These scenarios were used to test the sensitivity of mean current densities to absolute values for movement costs and the range of costs. Correlations were calculated among the same 1000 randomly selected cells within pairs of movement cost scenarios using two study areas: a) east coast provinces and b) southern British Columbia. (DOCX) [file pone.0281980.s002.docx]

| **Scenario** | **Low** | **Medium Low** | **Medium High** | **High** | **Range** |
| --- | --- | --- | --- | --- | --- |
| C1 | 0.1 | 0.5 | 1 | 1.5 | 1.4 |
| C2 | 1 | 1.5 | 2.25 | 3.375 | 2.375 |
| C3 | 1 | 1.5 | 2.25 | 225 | 224 |
| C4 | 1 | 2 | 3 | 4 | 3 |
| C5 | 1 | 2 | 3 | 300 | 299 |
| C6 | 1 | 5 | 7.5 | 10 | 9 |
| C7 | 1 | 5 | 7.5 | 750 | 649 |
| C8 | 1 | 100 | 150 | 200 | 199 |
| C9 | 1 | 10 | 100 | 1000 | 999 |
| C10 | 10 | 100 | 1000 | 10000 | 9990 |
