# Supplementary material for: Predicting areas important for ecological connectivity throughout Canada
Source: PLoS One. 2023 Feb 22;18(2):e0281980. doi: 10.1371/journal.pone.0281980 (PMC9946242; doi:10.1371/journal.pone.0281980)
Supplement: S3 Table — (DOCX) [file pone.0281980.s003.docx]

|  | **Grey Wolf Model** | | | **Mountain Caribou Model** | | |
| --- | --- | --- | --- | --- | --- | --- |
| *Predictors* | *Estimates* | *CI* | *p* | *Estimates* | *CI* | *p* |
| Intercept (Case = Available) | 1.9546 | 1.7646 – 2.1446 | **<0.001** | 1.3907 | 1.2737 – 1.5078 | **<0.001** |
| Case = Observed | 0.4402 | 0.3545 – 0.5260 | **<0.001** | 0.0398 | 0.0114 – 0.0682 | 0.006 |
| Individual Max Displacement | -0.0019 | -0.0037 – -0.0002 | 0.033 | -0.0012 | -0.0044 – 0.0020 | 0.457 |
| Case*Individual Max Displacement | 0.0038 | 0.0030 – 0.0045 | **<0.001** | 0.0049 | 0.0042 – 0.0056 | **<0.001** |
| N | 68 _id_ |  |  | 186 _id_ |  |  |
| Observations | 34892 | | | 49090 | | |
| Marginal R^2^ / Conditional R^2^ | 0.039 / 0.075 | | | 0.019 / 0.210 | | |
|  |  |  |  |  |  |  |
|  |  |  |  |  |  |  |
|  | **Moose Model** | | | **Rocky Mountain Elk Model** | | |
| *Predictors* | *Estimates* | *CI* | *p* | *Estimates* | *CI* | *p* |
| Intercept (Case = Available) | 2.111 | 1.5149 – 2.7071 | **<0.001** | 3.1844 | 3.0851 – 3.2837 | **<0.001** |
| Case = Observed | 0.0276 | -0.0864 – 0.1417 | 0.635 | -0.5208 | -0.5496 – -0.4920 | **<0.001** |
| Individual Max Displacement | -0.0003 | -0.0212 – 0.0206 | 0.977 | -0.0307 | -0.0344 – -0.0269 | **<0.001** |
| Case*Individual Max Displacement | 0.026 | 0.0224 – 0.0296 | **<0.001** | 0.0209 | 0.0197 – 0.0220 | **<0.001** |
| N | 19 _id_ |  |  | 175 _id_ |  |  |
| Observations | 11032 | | | 317096 | | |
| Marginal R^2^ / Conditional R^2^ | 0.063 / 0.22 | | | 0.019 / 0.043 | | |
