# Supplementary figures and images for: Predicting areas important for ecological connectivity throughout Canada
Source: PLoS One. 2023 Feb 22;18(2):e0281980. doi: 10.1371/journal.pone.0281980 (PMC9946242; doi:10.1371/journal.pone.0281980)

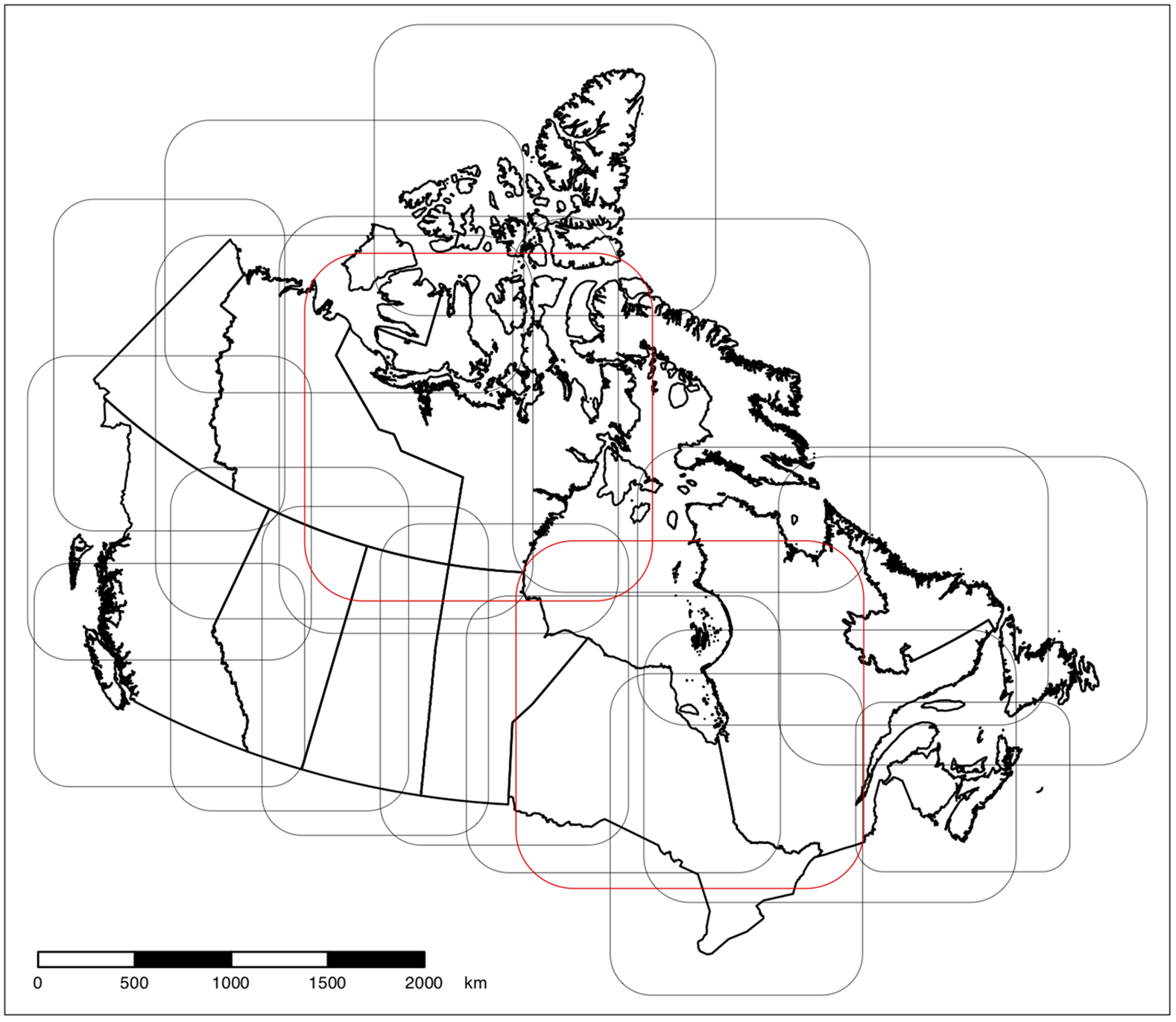

Supplement: S1 Fig — Black boxes indicate the 17 initial tiles used and red boxes the two additional tiles that were required to address anomalies at the seams. Contains information licensed under the Open Government Licence–Canada. (TIF) [file pone.0281980.s004.tif]

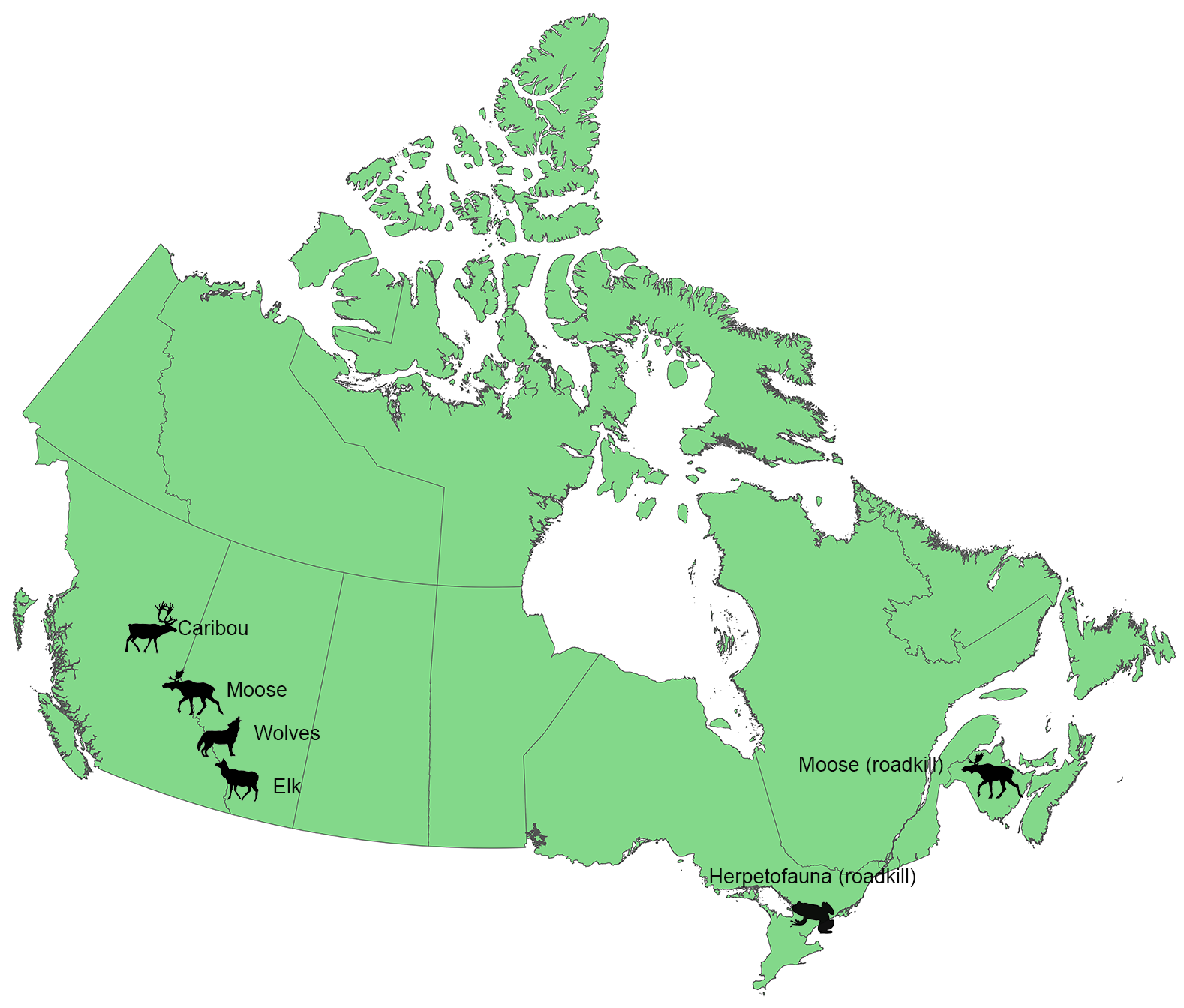

Supplement: S2 Fig — The data included GPS collar data for caribou in British Columbia, moose, wolf, and elk in Alberta; herpetofauna roadkill in Ontario, and moose roadkill in New Brunswick. Contains information licensed under the Open Government Licence–Canada. Public domain animal silhouettes were downloaded from https://beta.phylopic.org. (TIF) [file pone.0281980.s005.tif]

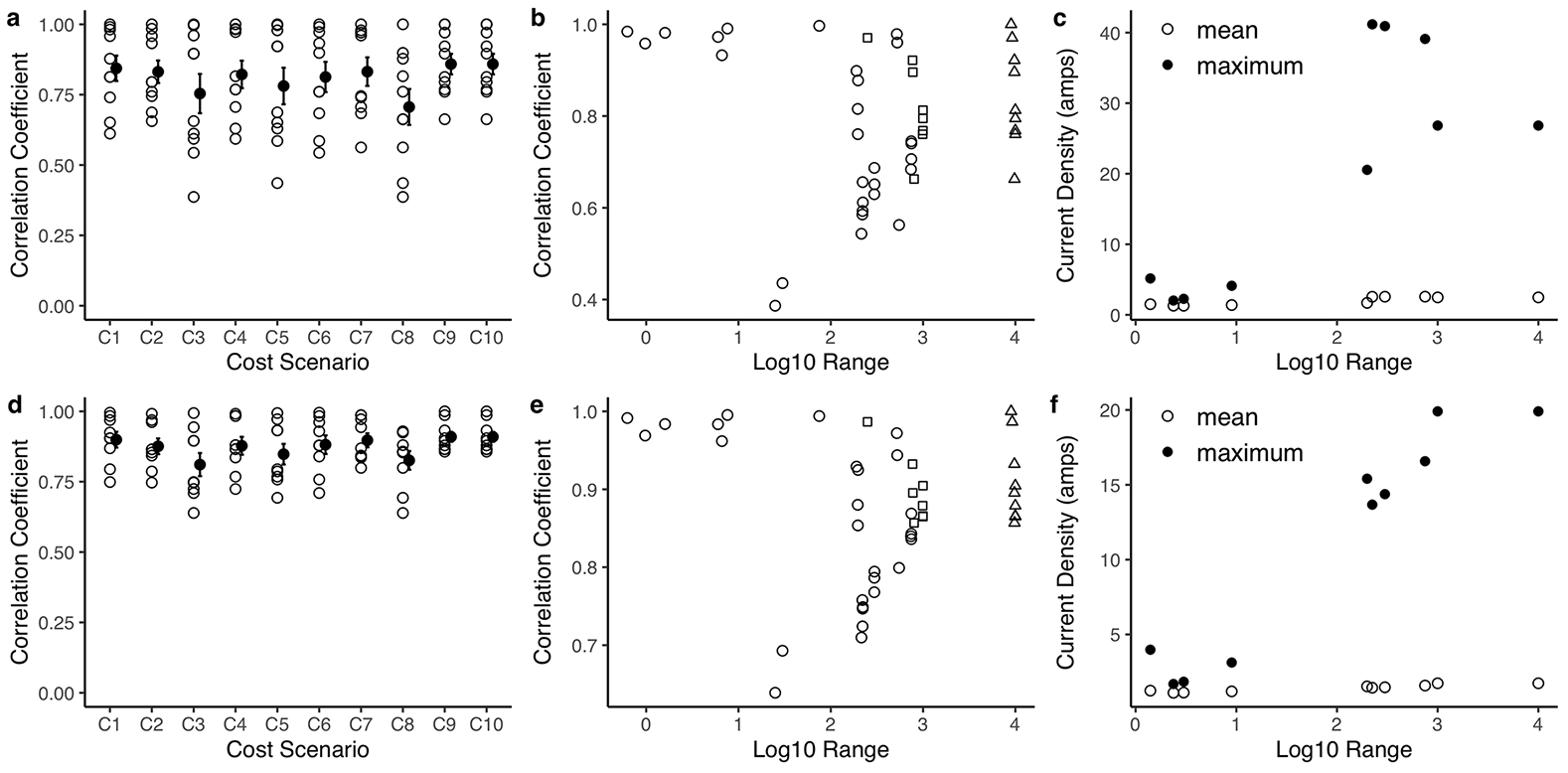

Supplement: S3 Fig — Correlations were calculated among the same 1000 randomly selected cells within pairs of movement cost scenarios using two study areas: a) east coast provinces and b) southern British Columbia. a) Correlations among pairs of cost value scenarios. Mean correlations were 0.79 and 0.84 for the two study areas, respectively. Scenarios 9 and 10 had highest mean correlation values with other scenarios (solid circles denote scenario means, and bars +/- one standard error. Open circles denote correlations of a given scenario with all other scenarios). b) Correlations arranged to display the effect of the range of cost values on current densities. This figure shows the effect of the absolute difference in the range of cost values between pairs of scenarios (log10-transformed) on the Spearman rank correlations. Pairs that include scenarios 9 or 10 are identified by square and triangle symbols, respectively. c) Effect of the range of cost values on current density estimates. Scenarios with broader ranges of costs (i.e., from low to high cost, log10-transformed) result in higher maximum current densities. (TIF) [file pone.0281980.s006.tif]

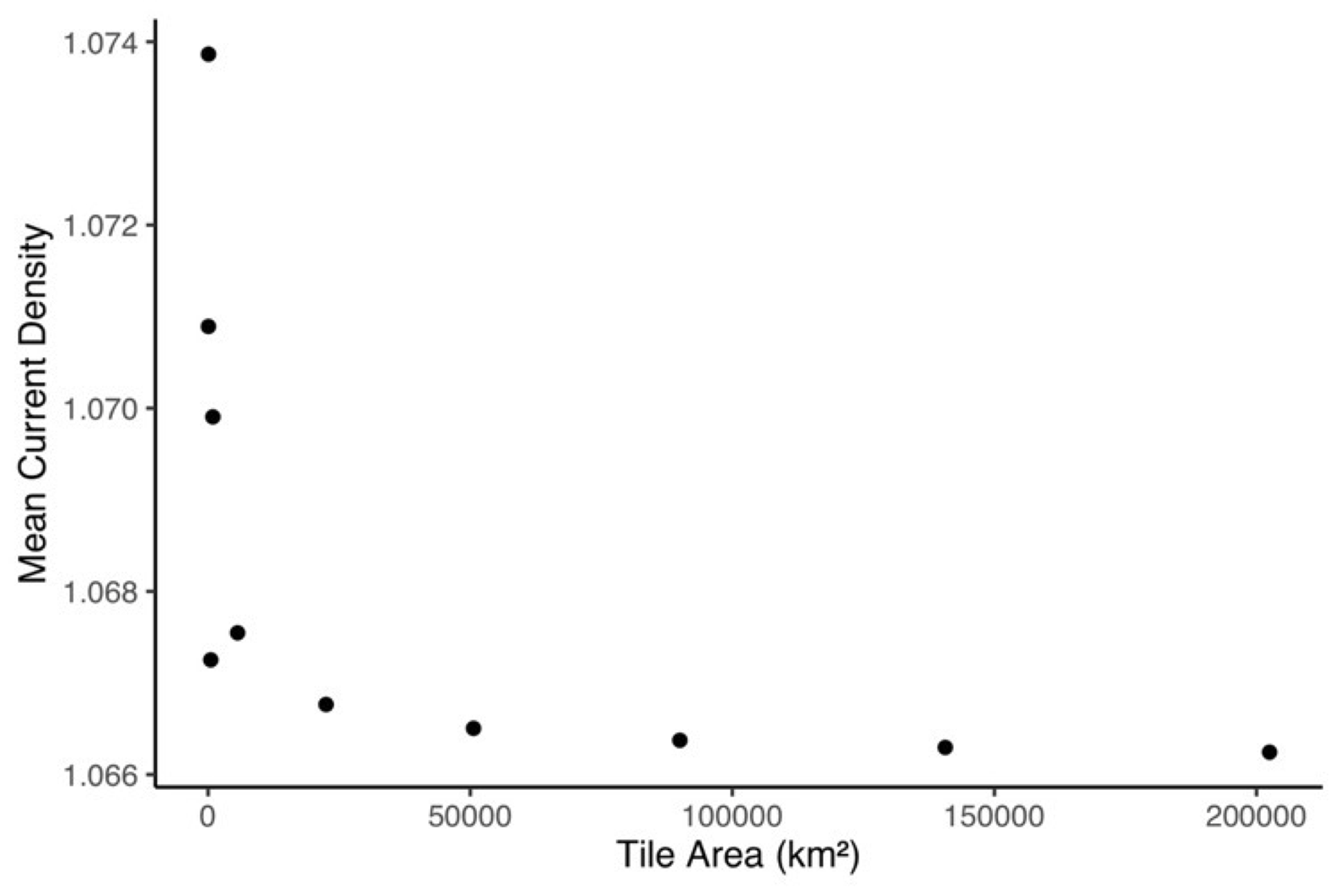

Supplement: S4 Fig — Analysis was conducted on simulated but identical landscapes, to control for composition and spatial distribution of cost values. The same pattern was found for the minimum, maximum, and standard deviation of current densities. (TIF) [file pone.0281980.s007.tif]

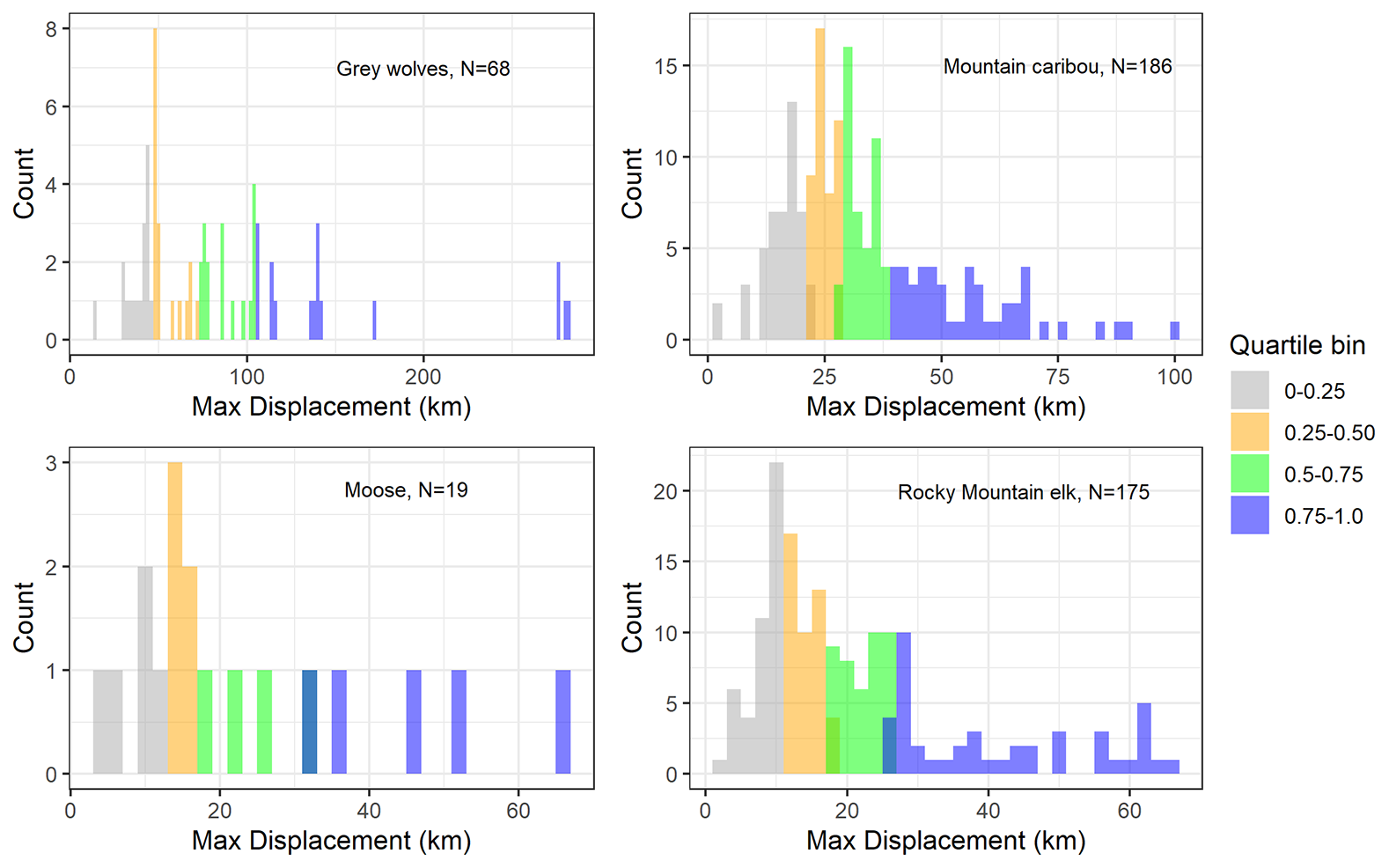

Supplement: S5 Fig — Counts are binned into quartiles, and colour coded. Maximum displacement is the distance from the first recorded location to the location recorded furthest away from that point. (TIF) [file pone.0281980.s008.tif]
