# Supplementary material for: Predicting areas important for ecological connectivity throughout Canada
Source: PLoS One. 2023 Feb 22;18(2):e0281980. doi: 10.1371/journal.pone.0281980 (PMC9946242; doi:10.1371/journal.pone.0281980)
Supplement: Attachment — Submitted filename: Response_to_Reviewers.docx [file pone.0281980.s009.docx]

**PONE-D-22-17312**

**Predicting Areas Important for Ecological Connectivity Throughout Canada**

**RESPONSE TO REVIEWERS**

**EDITOR’S COMMENTS:**

**1.** Please consider and reflect on all points raised by the reviewers (especially noting that reviewer 2 raised a number of concerns on the approach).

Response 1: We thank the reviewers for taking the time to provide comprehensive reviews; all three raised some very good points. We used their suggestions to revise what we think is a much improved manuscript. We followed Reviewer #3’s suggestion to emphasize the “focus on this first pan-Canadian, multi-species connectivity modeling exercise” and de-emphasize the methodological tests (e.g., sensitivity analyses) to the extent possible, while still addressing Reviewer #2’s concerns about the methods. We also clarified that we consider our work to be a test of the hypothesis that the connectivity of multiple species can be predicted using an ‘upstream’ modelling approach, to address other points raised by the reviewers.

**2.** If applicable, we recommend that you deposit your laboratory protocols in protocols.io to enhance the reproducibility of your results.

Response 2: All coding and maps will be made available to the public in OSF upon acceptance of the manuscript (https://osf.io/z2qs3/; DOI 10.17605/OSF.IO/Z2QS3.

**3.** We noticed you have some minor occurrence of overlapping text [in your abstract] with the following previous publication, which needs to be addressed: <https://www.researchgate.net/publication/342733129_Validation_of_functional_connectivity_modeling_The_Achilles'_heel_of_landscape_connectivity_mapping>

Response 3: Thank you for bringing that to our attention. We were not able to find any overlapping text (using Compare in MS Word) but our abstract has since been revised substantially.

**4.** In your revision ensure you cite all your sources (including your own works), and quote or rephrase any duplicated text outside the methods section. Further consideration is dependent on these concerns being addressed.

Response 4: We believe we have addressed any issues in the revised manuscript.

**5.** We note that Figures 4, 5, 6, 7, S1, S3 and S4 in your submission contain [map/satellite] images which may be copyrighted.

Response 5: We assume you were referring to the provincial boundaries used in the above-mentioned figures. These shapefiles are licensed under an Open Government License – Canada, which permits users to copy, modify, publish, translate, adapt, or distribute provided the users include an attribution statement acknowledging the source. We have now included an attribution statement within each figure caption where appropriate.

**REVIEWER #1:**

**1.** Movement cost surface (line 172): “In this study, we used both empirical data and expert opinion to construct a movement cost surface for Canada.” Could you please provide details on how expert opinion influenced the cost surface layer, what species this can/is used for, and how the experts determined the values feeding into this study?

Response 1:

We have added the following sentence to the methods to help clarify our use of expert opinion:

- “We ranked and assigned cost values to the anthropogenic layers using our own knowledge but in consultation with experts (see Supporting Information) and consistent with previously published studies [6,17–20,22,26,31–34].” Lines 146-149

We now cite the experts consulted in the Supplemental Information under Table 1:

- Members of the Canadian Connectivity Working Group (<https://www.conservation2020canada.ca/connectivity>), Jodi Hilty, Clayton Lamb

Our manuscript indicated which species our study was focussed on in this sentence:

- “Much like Koen et al. [18], we modelled connectivity for terrestrial, non-volant fauna that use and move across natural land cover more successfully than through anthropogenic land cover types.” Line 143 - 146

We have also added more citations for studies that found evidence that many species are more likely to use and move successfully through natural land cover types and conversely, avoid areas with higher degrees of anthropogenic impact.

**2.** Line 213. You mention consultation with experts. Please identify them and provide more details on what information you gathered from them and how.

Response 2: The experts that were consulted are now identified in the supplemental information. We presented our study to members of the Connectivity Working Group, which includes connectivity experts from academia, governments, and conservation groups from across Canada.

Movement costs were assigned to natural features based on published data, which are cited in the manuscript (and the supplemental information), and advice from Jody Hilty & Clayton Lamb. We used our own judgement to select specific thresholds for elevation and slope from among those available in publications, as they are different for different species. All cost values were presented to the Connectivity Working Group for review.

**3.** Circuit theory analysis: Are there ways you could quantify alignment of estimates from overlapping tiles? I am thinking of something along the lines of what folks at Cornell are doing with their AdaSTEM models (e.g. Fink et al. 2020). Not the modelling approach itself, but they way they eventually combine tile or stixel (as they call them) estimates into seamless layers.

Response 3: Yes, that is one way. Another would be the same way we conducted the sensitivity analysis of cost values, using Spearman rank correlations to compare the generated current density values for the same randomly selected cells within the two overlap areas. We have since used this approach and found a mean correlation of 0.78 and a range of 0.62 to 0.93 for overlapping areas. Note however that we rarely had overlapping tiles, because we trimmed the tile buffers off prior to stitching. We used buffers specifically to address biases in current densities due to node placement.

**4.** Fig 1. I am not clear on what the open circles represent. Could you clarify that in the figure legend? Do the open circles represent the other cost scenarios? I think they do, by am not entirely sure.

Response 4: Indeed, open circles represent the correlation of a given scenario with all other cost scenarios. We have clarified this in the figure caption in what is now S2a in the Supporting Information.

**5.** Reading the corresponding text in the results section I am also not clear why you chose C9. What makes it stand out compared to C1? Could you please provide more detail on your choice and how you chose?

Response 5: It is indeed difficult to see how cost scenario C9 stands out. We have added the word “all” to the sentence that says “We found that the current densities generated using ten cost scenarios were ALL highly correlated …” This supports the point that the absolute costs assigned are not overly important. However, we had to select one cost scenario. C9 & C10 had the highest correlation with all other scenarios, but C9 included a low cost of 1, which has been more conventionally used in previous studies.

**6.** Fig. 4 There is quite the contrast in cost values between Canada and the US apparent in Manitoba and moving West. Could you discuss possible implications of that and what causes this difference as well as why you did not try to align datasets across the border more?

Response 6: Koen et al. (2010) showed that random data can be employed in a buffer to account for edge effects caused by random walks near the map boundary. Random data in a buffer produced results inside the mapped area that were nearly identical to accurate map data. This arises because the role of the buffer is simply to allow space for random walks to occur rather than have them pile up on the map edge. This has become a common approach in circuit theory modelling where map edges come into play. Note also that we do not seek to quantitatively compare layers inside and outside of Canada.

**7.** Also, a red- green color ramp is hard for color blind people to decipher. Could you change the color ramp to something that’s color blind friendly?

Response 7: Yes, we have modified this figure as well as the vignettes to be colour blind friendly.

**8.** Fig. 5. To me this is the main result and figure of this manuscript. I would recommend providing more detail on what readers see on the map in the figure caption. It would be great if the figure (plus caption) could almost be a stand alone product that interested readers could look at and understand easily without needing to read much of the manuscript text.

Response 8: Good point. We have expanded the figure caption so it could be used as a stand-along product.

**9.** Validation results: I think the figure numbering is off in this section. I don’t think Fig. 7 shows the validation results for GPS-collar data. If I interpret Fig. 8 correctly, this information is included there. Is that right?

Response 9: Yes, thank you. This has been corrected.

**10.** I personally would not go as far as suggest these maps could be used at ‘local-scales’. Applying a Canada wide dataset to local scale connectivity work might be going a bit too far. Regional or landscape scale I can see, but given the input data used for this analysis, I would recommend avoiding ‘local-scales’ analysis as to not give the impression a small land trust could use this information for their planning spanning a couple quarter-sections in say Alberta.

Response 10: Agreed. We have changed the wording from local to regional and inserted this sentence in the discussion: “We believe the current density map can also be used to identify areas important for functional connectivity within regional initiatives, but we recognize the spatial resolution is likely not fine enough to make decisions on specific land parcels.” Line 511

**11.** Line 444 “highlight connectivity ‘pinch points’”. I think this is a very important point. Could you discuss the potential consequences of this effect on the results and their interpretation overall?

Response 10: We have expanded the discussion in starting at line 513.

**12.** Line 450+. In this paragraph you mention “We believe the resolution of our map is sufficient however, to support land management decisions and planning at local-scales, ..” As mentioned above, I find this framing a bit misleading. I agree that results at the resolution you are using for your map would work for local-scale planning, but I don’t think that all the input data you have used would allow for that. This is the same problem we face in our work at NCC. Our group provides national scale information for regional planning as much as we can, but we do acknowledge and point out that national scale data most often don’t lend themselves well for local-scale planning due to trade-offs between data granularity and spatial scope. That’s a main reason why one of your co-authors (KH-P) set out to refine the global human footprint to Canada. A person in the same group is currently working on refining these data further for use in the province of British Columbia. I would really like to see this important issue discussed in this manuscript.

Response 12: Agreed. Please see response 10 above.

**13.** Line 519+ “Validation of Results”: I very much like your validation of results section and how you used independently collected wildlife data in this manuscript. One thing I am wondering about though is whether correlation indicates causation though? Have you considered including some thoughts on other factors that could contribute to the correlation or how the ‘pinch points’ you mentioned earlier could be correlated with the results you found for herpetofauna?

Response 13: Thank you. We have made it more clear in the revision that the current densities are model predictions that we have tested with external data. We have now included some discussion about factors other than costs affecting current density. Please see the paragraph starting at line 513.

**14.** Line 557+ “Potential limitations”: Thanks very much for including this section. I think its very important that we all discuss potential limitations in our papers. This could be a good place to discuss both the ‘local-scales’ and ‘pinch points’ components that I mentioned earlier in case you don’t think it appropriate to discuss these earlier. I personally think both should ideally be discusses earlier in the text given their importance, but at the very least they should be discussed in this section.

Response 14: Good advice. See our responses above for #11 - 13.

**15.** I can’t access the data for review as the OSF repository is currently set to private. Depending on what can be found in the OSF repository the following paragraph might not apply here. Finally, I would like to include a request to the authors to make their data and code as available as reasonable. I’m a big believer in the FAIR (Findable, Accessible, Interoperable and Reusable) principle for scientific data management and stewardship (e.g. https://www.nature.com/articles/sdata201618), which is why I include a request like this in all my reviews. As an example: my questions related to the statistical tests used and how they were applied could have been answered by looking at the code used in this study. Others could also build on your work once its published.

Response 15: An excellent point. Our intention is to make our OSF site public upon acceptance of the manuscript. It will include our input cost surface and output current density maps, as well as the coding used for the analyses.

**REVIEWER #2:**

I see the following major issues, the first with the input, and the second with the model choice:

**1.** The authors claim that they use expert opinion and empirical data to generate cost surfaces. However, their description only points to expert opinion being used for generating costs surfaces. This is highly problematic.

a. The cost surface is the input parameter for connectivity models. They are important, to link species ecology to models, and to ensure that models have realism and utility for on-ground applications. In the past, expert opinion has been widely used, but this practice is fading with studies pointing out the issues with using expert opinion to parameterise models. Essentially, the study moves from a multi-species perspective (as per the authors' claim), to a study that does not consider species information.
b. All inference on natural barriers etc. to connectivity needs to be removed, as this is simply the input parameters provided by experts into the model.

Response 1:

a) There are two general types of multi-species connectivity models, those that integrate needs of multiple species at the outset of the analysis (called ‘upstream’ methods), and those that integrate multiple species needs at the end of the analysis (‘downstream’ methods) (Woods et al. 2022). For both cases, but especially the former, it is important to validate the models using external data. We have taken the approach of testing the hypothesis that we can use an upstream approach based on naturalness to efficiently model terrestrial connectivity for multiple species. We test this hypothesis using external data. We have tried to more clearly explain this approach and rationale in the introduction of our revised manuscript at lines 67 – 90 and 123 - 124.

b) The reviewer raises a good point that our description of current flow throughout Canada is not surprising, given how the input movement cost was parameterized, including for natural barriers. However, we believe it reflects real impediments to movement and therefore can assist in identifying areas important for connectivity. Nevertheless, we have clarified and reduced our discussion about the patterns of current density to address the concern.

Citation: Woods et al. 2022. Frontiers in Ecology and Evolution doi:10.3389/fevo.2022.830822.

**2.** I do not understand why the authors use an omnidirectional approach. Connectivity deals with linkages from a source to a destination. The two ends are important and are significant. You cannot have connectivity as a cul-de-sac – going from somewhere to nowhere, or from nowhere to nowhere. Yet, that is what the authors do in their paper.

- There is a pattern that comes about because habitat is placed the way it is in a landscape. By removing that pattern, you are removing some of the realism of connectivity model and disconnecting it from your landscape.
- As a result, the findings of the paper largely relate to identifying good quality habitats for biodiversity. I have a feeling the validation of mammalian herbivores is also reflecting the same patters.
- I’d strongly suggest modelling connectivity from identified source/destination populations/habitats in the landscape. I am sure the authors have access to this information.
- If, like in the northern reaches, there is no clear distinction and animals use the entirety of the landscape, then connectivity is not an issue in this landscape.

Response 2: We have made sure to clarify our rationale in the introduction for using an omnidirectional approach (lines 111-121). We also cite several papers that use and explain the value of omnidirectional connectivity methods (e.g., Koen et al. 2014, Pelletier et al. 2014, McRae et al. 2016, Phillips et al. 2021). Additional papers are cited below. Briefly, while source and destination nodes are important for connectivity analyses, they are not always known a priori. Furthermore, current density patterns are biased by the placement of nodes (i.e., the same landscape will produce widely differing outputs depending on node placement) (Koen et al. 2014). Consequently, we consider an omnidirectional approach the best method to produce a seamless, multi-species current density map that is not dependent on node placement. We think these considerations are especially important in the context of a large-scale approach to mapping potential connectivity for the purposes of land-use planning.

The reviewer suggests that “the findings of the paper largely relate to identifying good quality habitats for biodiversity”. We note that Koen et al. (2014) found that current density estimates from an omnidirectional circuit theory model were a better fit to movement data for a variety of taxa than a simple cost surface (input) map, demonstrating that the map is something more than a habitat map. This is evident in our map by the number of places where high current density occurs in high-cost areas. These ‘pinch points’ are disproportionately important areas for movement due to their proximity to habitat, and other aspects of the geometry of the landscape.

Omnidirectional connectivity methods were described in PLOS One (Pelletier et al. 2014) and in Methods in Ecology and Evolution (Koen et al. 2014) and have since been used in numerous other studies, including:

- Hohbein RR, Nibbelink NP. Omnidirectional connectivity for the Andean bear (Tremarctos ornatus) across the Colombian Andes. Landscape Ecology. 2021 Nov;36(11):3169-85.
- Choe H, Thorne JH. Omnidirectional connectivity of urban open spaces provides context for local government redevelopment plans. Landscape and Ecological Engineering. 2019 Jul;15(3):245-51.
- Gray ME, Dickson BG, Nussear KE, Esque TC, Chang T. A range‐wide model of contemporary, omnidirectional connectivity for the threatened Mojave desert tortoise. Ecosphere. 2019 Sep;10(9):e02847.
- McClure ML, Dickson BG, Nicholson KL. Modeling connectivity to identify current and future anthropogenic barriers to movement of large carnivores: a case study in the American Southwest. Ecology and evolution. 2017 Jun;7(11):3762-72.
- Fleishman E, Anderson J, Dickson BG. Single-species and multiple-species connectivity models for large mammals on the Navajo nation. Western North American Naturalist. 2017 Jul;77(2):237-51.
- Borja-Martínez G, Tapia-Flores D, Shafer A, Vázquez-Domínguez E. Highland forest’s environmental complexity drives landscape genomics and connectivity of the rodent Peromyscus melanotis. Landscape Ecology. 2022 Mar 15:1-9.

**3.** Lines 44-62: This is a lot of text to state that connectivity is important and can be condensed to focus on the goal of this particular exercise. In that sense, while climate change mitigation and migratory species conservation is also relevant, I get the sense that maintaining connectivity across populations and habitats of species is the primary concern here?

Response 3: We have shortened the background information.

**4.** Lines 69-82: I am not sure I understand this distinction. Are you suggesting there are two approaches: one that uses species information, and collates resulting maps; the other that uses a sort of least-common-denominator of species movement, i.e., the assumption that species will be impacted by human presence and ignores all other species traits.

Response 4. In general, yes. We have revised the introduction to clarify these points, as described in response 1 above about upstream and downstream approaches to multi-species connectivity modelling. The topic has been explored in detail recently by Woods et al. (2022).

Citation: Woods et al. 2022. Frontiers in Ecology and Evolution doi:10.3389/fevo.2022.830822.

**5.** Given that, as you say in lines 67-68, species “differ in their movement traits, resource needs and sensitivity to human disturbance”, how is this least-common-denominator approach tenable in the face of its mismatch with this theoretical knowledge?

Response 5. We also state that “the movement patterns of many species, for example, are known to be negatively affected by human-modified land cover features such as cities and roads.” That knowledge can be used for upstream approaches to multi-species connectivity modelling, as described in our previous response 1. Our objectives, therefore, included testing the hypothesis that we could use an ‘upstream’ approach based on naturalness to model multi-species connectivity. We consider this to be an efficient, and potentially effective method for producing a seamless connectivity map at a large-scale (e.g., at a national level). Importantly, determining effectiveness depends on validation for a variety of species, which we have started in the present manuscript but plan to continue. The alternative (downstream) method of producing and combining species-specific connectivity models is complicated for a variety of reasons, not least of which is that there is no common set of species that occur across all of Canada. We consider the approach we have taken to be a potential solution for practical, applied land-use planning across a national extent.

**6.** Lines 88-98: You can condense this; Circuit theory based modelling has been in use for a while now.

Response 6. Condensed as suggested.

**7.** Also, take a look at: van Moorter et al 2021 Ecography, DOI: 10.1111/ecog.05351, and Panzacchi et al. 2016, J Appa Ecol. DOI: 10.1111/1365-2656.12386. There is a new approach to modelling animal movement, called Randomised Shortest Path. You may consider using that approach.

Response 7. Thank you for the suggestion. We agree that is an important new approach and have now cited those papers in the revised manuscript. The van Moorter paper illustrates the challenge of analysing ‘effective connectivity’ for multiple species, noting how much data is required in Table 1.

**8.** Lines 108-110: Under what conditions would you want to model connectivity in general, as opposed to among specific locations? From your introduction, I understood that you wanted to model connectivity among species habitat patches (specific locations).

Response 8. We have revised the text here to clarify our interest in modeling connectivity in general (lines 108 – 121 and 130 - 132). We simply intended that source and destination sites are not always known, especially for multiple species in a national analysis.

**9.** Lines 111-112: Why are sources and destinations unknown here? Also, I would disagree that the omnidirectional approach assumes uncertainty in sources and destinations. It does carry an assumption about sources and destinations; I think there is some lack of clarity about what this assumption is (locations at the edge of the chosen landscape are sources and destinations?), and this lack of clarity is a problem. Please clarify.

Response 9. It is not that source and destination sites are necessarily unknown (although for our study that is the case); rather, a key objective of omnidirectional methods is that the resulting output does not *depend* on source and destination node placement. The methods have been developed so that the results are independent of nodes. A map produced through omnidirectional methods is therefore generally applicable to a landscape in the absence of knowledge about particular source and destination scenarios. This is important in the context of providing advice for applied land-use planning, where details about node placement may vary from application to application. We have revised the wording here (lines 108 – 121 and 130 - 132) to clarify this point.

**10.** Line 112: What is generalised land use planning activities? There has been a lot of movement towards land use planning that takes into account ecological information on species and ecosystem requirements. If generalised planning activities are those that ignore these requirements, then the use of these is incongruent with advanced movement models, aren't they? Further, I'd note, the solution to the challenge of combining information on multiple species is not to neglect inclusion of such information.

Response 10. Please see our response 5 above. As an example of generalized land use planning, in Canada, land-use planning is largely a provincial or territorial jurisdiction. For example, in the Canadian province of Ontario, there is legislation that requires land-use planning based on naturalness, without reference to particular species. Further, each Canadian province and territory has their own land-use planning legislation. There are also no terrestrial species with distributions that cover the entire country, supporting the development of a seamless ‘downstream’ map. There is also only movement data available for a small proportion of the potential species that could be relevant for such an approach. Nevertheless, Canada also has several federal initiatives mandating connectivity assessments at a national level. Given these various considerations, we considered it an appropriate step to test the hypothesis that we could use an upstream approach based on naturalness to develop a seamless map at a national extent for the purposes of supporting land-use planning across this large, diverse region. Validation of our model with a variety of empirical movement data is an important aspect of this approach. We have added some material to the introduction to make the rationale for a national analysis more clear.

**11.** Line 113: [Omniscape] It has become available, but why is it useful?

Response 11. We have removed this reference to Omniscape.

**12.** Lines 122-124: I found this finding interesting and surprising, so I went through the paper. The overall correlations may be high, but the locations of high-movement in the cited paper across different relative cost categories changes. This matches with findings from Rayfield et al. 2010, Land.Ecol. DOI: 10.1007/s10980-009-9436-7, who found that least-cost-paths were sensitive, not to absolute values of costs, but to their relative costs. So, assigns costs are important and need considerable thought.

Response 12. We are unsure of what the reviewer means here, but the Bowman et al. (2020, PeerJ) manuscript did not show that “the locations of high-movement in the cited paper across different relative cost categories changes”. In fact, there was a lot of agreement among all of the cost scenarios, as shown by the high correlations, provided that the rank order of the costs was maintained. Rayfield et al. (2010) found that route placement in least-cost paths is sensitive to fragmentation, and Bowman et al. (2020) did find an analogous result in that when “the rank order of costs was not maintained, by changing the spatial arrangement of the costs, correlations were reduced”. In other words, so long as the rank order of the costs is maintained, the current density outputs are highly correlated. Current density values are sensitive to rank order of costs, but not to absolute costs. We found the same pattern in the sensitivity analyses conducted in support of our manuscript. We have clarified these points in the revised manuscript.

**13.** Line 129: By themes, you mean the number of factors influencing resistance? Why is this?

Response 13. Yes, we used ‘themes’ as a shorter version of ‘thematic resolution’, or the number of land cover types used in the analysis. Analyses that include more cover types will likely have more uncertainty, as the data usually does not exist for parameterizing a model to reflect the movement of multiple species across several land cover types. Instead, we used the approach of generalizing land cover and using only four movement cost values (1, 10, 100, 1000). For example, we feel more confident to assign the same cost value of 1000 to ‘built environments’ and ‘oil and gas’ as opposed to estimating whether one or the other should be assigned a higher cost value. However, that particular sentence in question has since been removed in an attempt to shorten and simplify the manuscript.

**14.** Line 130: There are two things to measure: the 'theme', and its influence on connectivity.

Response 14. This sentence has been removed to shorten and simplify the manuscript. However, as mentioned above in response 13, we used ‘themes’ as a shorter version of ‘thematic resolution’, or the number of land cover types used in the analysis.

**15.** Line 142-143: 'only amongst mostly large protected areas' -- is your contention that they missed a significant proportion of animal habitats or populations?

Response 15. Across Canada, only 12.5% of the land is currently included in the network of protected areas. We wished to develop a seamless map that accounted for the potential contribution of all sites to connectivity, and that was not influenced by the starting point of the existing protected areas network. In Canada, there are many natural areas that contribute to connectivity outside of the PA network.

**16.** Lines 144-145: True, but depending on the species, some of them are confined -- in terms of viable source populations -- to PAs. The utility of this is really dependent on the species in question.

Response 16. We agree that focussing on viable source populations is an important approach for modelling connectivity and may be appropriate for some countries. However, as mentioned in the manuscript and response 15, only 12.5% of Canada’s terrestrial areas are protected and there are many natural areas outside of protected areas supporting viable populations. In addition, most protected areas are not large enough to support viable populations for many species.

**17.** Lines 133-146: Why is it important to get this at the national level? This is a huge nation and likely larger than the dispersal range of most species. Likely encompasses multiple conservation landscapes. I am sure there are species that are restricted in range such that they are not present across the country. So what is the relevance or significance or utility even, for a country-wide cross-species connectivity modelling exercise?

Response 17. We have added some text to the introduction and discussion to clarify our rationale for developing a national map. Please also see our response 10 above. Some of us are directly involved in federal and provincial programs that are already using our current density map.

**18.** Lines 172-173: If you have empirical data, why use expert opinion? Expert opinion has not stood well, nor is it scientifically justifiable, for generating model input parameters.

Response 18. We note in the manuscript that this is unrealistic for a map of an area as large and diverse as the country of Canada, which is why we tested an upstream approach using a generalized movement cost surface. Importantly, we validate the results of our analyses with independent data, an important step that is rarely done even for single species studies. We provide further explanations in our responses above to your comments #1 and #12.

**19.** Lines 174-175: A clarification: You used only those fauna that used natural land uses more, or you found that the majority of fauna used natural land uses more?

Response 19. We have clarified this in the manuscript. Lines 143-149 and 248-249:

- “Much like Koen et al. [18], we modelled connectivity for terrestrial, non-volant fauna that use and move across natural land cover more successfully than through anthropogenic land cover types by assigning higher costs to land covers with higher degrees of anthropogenic disturbance”
- “Consequently, we did not include data for peri-urban species (e.g., raccoons, skunks, coyotes)” Line 249

**20.** If the former, how did you form criteria for inclusion in your analyses?

Response 20. We have clarified this in the manuscript (see above response 19). Furthermore, we used all of the independent data we could acquire in time for this study. Some researchers that we contacted suggested their data will be available in the future once they complete their own studies.

**21.** Lines 175-176: I don't understand this sentence.

Response 21. We have expanded our description of the upstream approach in the introduction.

**22.** Line 183: I don't understand this. You used a buffer outside Canada, and different layers for that buffer? Won't that pose problems in terms of how compatible and directly comparable the layers inside and outside Canada are?

Response 22. Koen et al. (2010) showed that random data can be employed in a buffer to account for edge effects caused by random walks near the map boundary. Random data in a buffer produced results inside the mapped area that were nearly identical to accurate map data. This arises because the role of the buffer is simply to allow space for random walks to occur rather than have them pile up on the map edge. This has become a common approach in circuit theory modelling where map edges come into play. Note also that we do not seek to quantitatively compare layers inside and outside of Canada.

**23.** Lines 194-197: Again, I'd point to the fact that relative rankings do not just mean the order, but the quantified relative costs across land uses.

Response 23. We have edited the text to include examples of rankings, absolute values, range of values. Lines 170-174

**24.** Lines 201-204: I am not sure this approach works. This seems like a pretty superficial comparison. Locations of movement are not all equally important -- pinch points for instance, are more important in terms of inference and prediction than other locations. A correlation coefficient masks these nuances. I'd suggest looking at this deeper in terms of where the variations in rank occur. And while I see why you take ranks -- I agree that the relative and not absolute values make a difference, consider this case: a landscape with large areas of medium movement, some of low movement and some of high movement. A landscape with clearly distinguished low and high movement locations -- rankings will not separate these two scenarios out. You may want to consider scaling current values to be on a comparable scale, instead of ranking.

Response 23. We prefer to use Spearman correlations in the manuscript because they are more robust than Pearson correlations to potential violations of spatial non-independence among the samples. However, we have also followed the reviewer’s suggestion here, and compared scenarios using mean-centred data (z scores) with Pearson correlations, which also demonstrated high correlations across all scenarios, supporting the conclusions we reached with the Spearman correlations (Maritimes: Spearman, mean r_s_ = 0.79, range = 0.39-1.0; Pearson of standardized values, mean r = 0.70, range = 0.25-1.0; BC: Spearman, r_s_ = 0.87, 0.63-1.0; Pearson, mean r = 0.79, 0.58-1.0). Therefore, after following the reviewer’s suggestion, we remain confident that current density tends to be highly correlated across cost scenarios provided that the rank order of the costs is maintained.

**24.** My second point: it is not the case that the scenario that correlates the most with others is the best scenario.

Response 24. We have clarified that this comparison was not our rationale for choosing a scenario. It was simply an effort to assess the sensitivity of our outputs to the choice of scenario. We conclude that the sensitivity is low.

**25.** Line 207: Assigned based on empirical data, you had mentioned. Could you please expand?

Response 25. Please see responses above to comments #1 and #2 from Reviewer #1

**26.** Lines 206-219: It seems to me that the costs were assigned based on expert opinion alone. I do not see any details here on how empirical data were used for assigning costs. If so, the authors should state this very clearly. And if so, this is a problem that needs to be addressed.

Response 26: Please see responses above to comments #1 and #2 from Reviewer #1.

**27.** Lines 239-241: Please expand on these terms. [wall-to-wall omnidirectional, Julia implementation, advanced mode of Circuitscape]

Response 27. The wall-to-wall method, which we used, is described in the methods starting in line 215. We have not included descriptions of the other two methods in part to address Reviewer #3’s request to reduce the content related to the methodological advances and because they are well described in the cited Phillips et al. paper.

**28.** Lines 243-245: Why is this useful? Taking a step back, how does it fit with dispersal theory? If we go by the assumption that connectivity links between two nodes -- i.e., the nodes are important, and you can't have a stairway to nowhere -- then, where does this mode of connectivity sit? Where is it useful and how does it compare (or not) with connectivity of species or ecosystems between specific nodes.

Response 28. This is the standard method of employing wall-to-wall omnidirectional circuit theory. The resulting output is a mean current density arising from nodes in all cardinal directions across the tile (i.e., a mean of random walks). The wall-to-wall method was initially developed and described in detail by Pelletier et al. (2014; cited 113 times, Google Scholar), and it has been compared to other omnidirectional methods by Phillips et al. (2021). Both of these papers are cited in the manuscript. We think that a detailed exploration of the theoretical underpinnings of the method are beyond the scope of our current paper, especially given that this is an established method that is described in the literature many times.

**29.** Lines 257-261: If mammalian herbivores and herpetofauna were the target species, why not formulate cost surfaces for these species/species groups?

Response 29. In the manuscript we state that “the movement patterns of many species, for example, are known to be negatively affected by human-modified land cover features such as cities and roads.” We used that knowledge to parameterize a cost surface for use in an upstream approach to multi-species connectivity modelling, as described in our previous responses 1, 5, and 10. Our objectives, therefore, included testing the hypothesis that we could use an ‘upstream’ approach based on naturalness to model multi-species connectivity.

**30.** Lines 263-273: Were these GPS collar data on dispersing animals or within their home ranges? Current density maps tend to light up 'habitats', due to their low cost. So, if these are of animals within populations / within their habitats, then it will generate high current values, but not because they are useful for movement - it is because they are habitat patches.

Response 30: Please see our response above to #2. Koen et al. (2014) demonstrated that a current density map is something more than a habitat map. In addition, thanks to you and reviewer #3, we reanalysed the data to include individuals as a random effect and ran linear mixed effects models for each species with displacement distance to examine differences between short and long-distance movers. Differences in current density at observed and available locations was greater for the long-distance movers compared to the short-distance movers (Fig 6 and S3 Table; and see S5 Fig for histograms of individual maximum displacement distances and their respective binning as short or long-distance movers).

**31.** Lines 295-297: These are effectively the same cost scenarios, unless you have some cells which are of lower (1) cost. This is because the relative costs (the ratio of costs of any one land use to another) remains the same across the two scenarios. Any difference between the two is likely due to randomness.

Response 31. Bowman et al. (2020, PeerJ, cited in the manuscript) show that the absolute range of cost values is positively related to maximum current density. We confirmed this with our data (S2c Fig in Supporting Information). Therefore, C10 would differ from C9 in producing a higher maximum current density value. We interpret that the higher contrast (i.e., wider range between low and high values) leads to an increased tendency for the creation of pinch points. Therefore, in addition to ratio of costs being important to current density, as the reviewer notes, the range of the cost values is also important.

**32.** Lines 311-312: This points to the fact that there are more 'pinch points' in these cost scenarios.

Response 32. We found that a higher range of costs (i.e., a greater difference between highest and lowest costs) was positively related to maximum current density. This was also observed by Bowman et al. (2020). (See also our previous response 31). This effect occurs because a higher contrast leads to more clearly defined pinch points, rather than necessarily more pinch points. Current will be less likely to spill into neighbouring pixels where the contrast in costs is high.

**33.** Lines 293-337: These results are difficult for me to follow or understand, or infer anything from as the cost scenarios are not meaningfully formulated; i.e., there is no ecological hypothesis, or linking to any of the cost scenarios. The paper would be hugely strengthened with this linking of animal ecology and the modelling.

Response 33. Please see our responses to your comments above (#1, 5, and 10) which address this concern. As explained in response 1, we have more clearly explained our approach and rationale in the introduction of our revised manuscript at lines 67 – 90 and 123 - 124.

**34.** Lines 354-356: But these were your input parameters, so it is not surprising that current densities will be influenced by these parameters?

Response 34. We have revised the wording.

**35.** Lines 367-368: Link this to my previous observation that current densities tend to be high in 'habitats'. This is just picking up suitable areas for animals to 'reside'; they do not necessarily also indicate good connectivity areas.

Response 35. In Response #2 above, we note that Koen et al. (2014) found that current density estimates from an omnidirectional circuit theory model were a better fit to movement data for a variety of taxa than a simple cost surface (input) map, demonstrating that the map is something more than a habitat map. In addition, we note that not all habitats have high current density, and not all areas of high current density are good habitats. This is evident from comparing the cost surface with the current density map. It is these discrepancies that we are particularly interested in when carrying out an omnidirectional connectivity analysis.

**36.** Lines 367-375: In my reading, this is why the omnidirectional connectivity that ignores known locations of importance for biodiversity (populations), is problematic. It highlights these known locations as important for movement, while they are in reality important habitat. To an extent, is also masks our inference on actual connectivity areas, because they may not show up as clearly as these habitats.

Response 36. Including the distribution of populations is relevant and helpful in cases where the population is discretely distributed, or where the distributions are known with some detail. However, in many applications of connectivity modelling for land-use applications, this information is either not known, is incompletely known, or the species are not discretely distributed. Given the set of options, we considered it efficient to take the approach of testing the hypothesis that we can use an upstream approach based on naturalness to efficiently model terrestrial connectivity for multiple species. We test this hypothesis using external data. Please see our responses 1, 5, 10, and 30 for more discussion of this important topic.

**37.** Lines 398--: How many animals were collared, and I hope you included animal ID as a random effect, or tested if this had an impact?

Response 37. Thanks to you and reviewer #3, we reanalysed the data to include individuals as a random effect and ran linear mixed effects models for each species with displacement distance to examine differences between short and long-distance movers. We have also added the numbers of individual animals to the manuscript.

**38.** Lines 426-428: Yes, but it was not parameterised by wildlife data.

Response 38. As we have noted, rather than parameterizing our model with wildlife data, we have taken the approach of testing the hypothesis that we can use an upstream approach based on naturalness to efficiently model terrestrial connectivity for multiple species. We test this hypothesis using external data.

**39.** Lines 433--: These are your assumptions going in, which came from expert opinions, and not data. I would remove all inference about this, unless you can back it with data.

Response 39. We have revised the wording here. Note that we used external data to validate our model.

**40.** Lines 445-448: This is a good point.

Response 40. Thank you.

**41.** Lines 490-492: Didn't you find that the high cost values were highlighted as the range of cost values increased (as mentioned in your next sentence)? That doesn't support this inference.

Response 41. Correlations across cost scenarios are generally high (e.g., mean Spearman rs across all scenarios in BC = 0.87, range = 0.63 - 1.0). Increasing the contrast in costs can reduce correlations through effects on maximum current density (as we have described in responses 23 and 31). However, even the lowest correlation was still positively correlated (rs = 0.63, between C3 and C8 with widely differing cost ranges). Therefore, we infer that current density has low sensitivity to varying costs if the rank order is maintained, but also that when there is an effect of varying costs it is largely due to the effect of the range of costs on maximum current density. We have clarified our inference in the text here by adding this latter point in lines 67 – 90 and 123 – 124.

**REVIEWER #3:**

**1)** General comment #1 – Abstract – intro - discussion: Although the authors are clearly recognizing that connectivity is a species-specific concept (line 67), it is unclear if they were modeling **structural or functional** connectivity. The only mention of functional connectivity is made at line 81 when referring to Koen’s et al. work. This is important, as many of the recent and novel connectivity research is made on functional connectivity, based on animal behaviour using expert knowledge or empirical data. I can understand that it was impossible to model functional connectivity at a community level over such a large study area, but recognizing the differences between structural and functional connectivity is important, at least in the introduction and discussion. In the discussion, clearly positioning the results along this structural – functional connectivity gradient would be interesting.

Response 1: We really appreciated your advice and have revised the manuscript following your suggestion in #3 below, reducing the emphasis on the methodological advances and instead emphasizing that our study was a test of the upstream approach for modelling connectivity for multiple species.

We believe the structural – functional gradient, coarse / fine filter approaches, and upstream / downstream approaches are inter-related with significant overlaps. E.g., the upstream approach requires the use of a cost surface that has to simplify and generalize land covers (as you point out below) in order to reflect the movement patterns of multiple species, and in doing so moves along the gradient from functional to structural connectivity. However, the employment of a movement model capturing an aspect of animal behaviour (i.e., a random walk) brings an aspect of functional connectivity to the analysis. This was further supported by the relationship between current density and independent estimates of animal movement. We have clarified some of these points in the discussion, including in lines 143 – 145.

**2)** Abstract (line 37): Could be interesting to briefly explain why the validation failed with herpetofauna in the abstract. We have to wait up to the end of the discussion to find out.

Response 2: Revised as suggested.

**3)** Introduction (general comment #2): Despite my general comment #1 above, I think that the introduction is quite long (5 ¼ pages). This is not that bad, but I consider that you’ve missed the opportunity to clearly focus on the “lack of knowledge” in the introduction, diluting this with many interesting, but not essential, information that can be moved to the methods (e.g. lines 119-131) or to the discussion (e.g. lines 55-62). This brings me to my second “general comment”: it is unclear in the introduction, as well as elsewhere in the manuscript, if it is a manuscript focusing on methodological advances/development or on ecological/conservation issues. This reflects into the structure of the introduction, with passages referring to the importance of connectivity for the ecology of wildlife in disturbed landscapes (lines 44-53), some statements referring to policy and land-use management (lines 55-62), followed by methodological background (lines 64-131), by the Canadian connectivity status (and importance) (lines 133-146), and finally by the objectives and brief overview of the work done (lines 148-160). Similar bi-modal structure appears in the Results section, with methodological findings (lines 293329, Figs 1-2-3) followed by ecological/landscape ecology results (lines 331-383, Figs 4-7), and back on methodological results (lines 396-414, Figs 8-9). While I can understand that the validation process is important for both methodological and ecological focuses, the first 2/3 of the results are equally treated, which is not essential and can confound the reader (I was questioning myself at several places while reading to figure out what was the real scope, and contribution, of this paper). Obviously, the same bi-modal structure was there in the Discussion too.

So my suggestion to the authors is the following: put front – from the introduction to the discussion – the primary focus of your paper. Is it a methodological paper, with clear demonstration that multispecies connectivity models can be built over such a large area, with this spatial resolution (tiles and grain), and with the different cost-scenarios and sensitivity analyses? If so, please diminish the length of the sections focusing on the importance for Canada land-use planning and conservation, from the intro to the discussion; keep some interesting sentences, but shorten these passages. However, I am not convinced this would be the best option, as you just applied and repeated work done by others (among which Jeff Bowman previous papers). So the best option would probably be to focus on this first pan-Canadian, multi-species connectivity modeling exercise, with all the pros and cons noted for the different species during the validation process. If this is the way you want to take, then you could shorten the intro by moving methodological stuff to the methods, shorten the results by sending several paragraphs and figures (1-2-3) to Supplementary material or Appendices, and shorten the discussion by focusing on ecological findings, while reassuring the readers with a short section on the application of the sensitivity analyses. Doing so, you’ll focus on (what I think is) the most important contribution of your paper, in a more balanced manuscript. I let this decision to you (and to the Editors), but this would result in a more interesting – and easier to read and understand – paper.

Response 3: Thank you very much for your suggestions. We agree that there were mixed storylines. We have taken your advice to heart and revised the manuscript to focus on the study being a test of the use of an upstream approach for modelling connectivity for multiple species.

**4)** Introduction (lines 148-151) : this is unclear to me what is a spatial grain “sufficient” to support local land management decisions. Could you give some more details?

Response 4: We have edited the manuscript and now use ‘regional’ instead of ‘local’ (in response to Reviewer #1) and we include a statement that says: “We believe the current density map can also be used to identify areas important for connectivity within regional initiatives, but we recognize the spatial resolution is likely not fine enough to make decisions on specific land parcels.” (Lines 509-511)

**5)** Methods (lines 172-173): This is great that you used both expert opinion and empirical data to construct a movement cost surface for the entire country. But please provide more details, as I wasn’t able to find where “expert opinion” came from, what it is, vs. empirical data. Also, please explain with more details why this is important to combine both source of information, and get back to that in the discussion; is it really a strength? See my main comment regarding the discussion below for more guidelines.

Response 5. Please see response to Reviewer #1, comments #1 & 2.

**6)** Methods (lines 173-177): so you are building a kind of hybrid between a structural and functional connectivity analysis, as you are oversimplifying the heterogeneity of the landcovers (wildlife habitats) for the studied species. This is very “coarse filter”, which is not a real problem considering the number of species modeled but also the impressive spatial scale (as well as the difficulty to gather and put together different sources of information). But this deserves more explanations in the discussion, especially contrasting this with other (one-species only, provincial, territorial, local) connectivity analyses. Again, see my main comment regarding the discussion below.

Response 6. Please see Response #1 above.

**7)** Methods (general comment #3): some decisions made in this section are not supported by (enough) peer-reviewed literature; you can strengthen the confidence of the readers by adding more support to the choice you have made while modeling it. Also, please pay attention to the explanation of the risks associated with the edge-effects (i.e. vignetage; see lines 108, 154, 183-188, 233, 247) while modeling on each tiles. You did great with your analyses, but I am not convinced that a non-initiated reader will fully understand “why” you did what you did.

Response 7. Thank you. If you are referring to how we assigned movement cost values, we have provided more information in the manuscript and supporting information, as mentioned in responses to Reviewer #1, comments #1 & 2 above. With respect to edge effects, we have added more text in the introduction (lines 111-121) to explain the effect of node placement and moved the use of buffers to the methods in lines 233-235, in part to shorten the introduction.

**8)** Methods (lines 260-261): Using road kills is not necessarily the best option to validate connectivity, as they occur on roads and roads are often considered as a (fine-scale) high-resistance feature. Could it be possible that the lack of support for the validation of your corridors using herpetofauna roadkill data be linked to the nature of your validation metrics (roadkills) more that to the space-use ecology (home-range size) patterns of these smaller species? This could deserve more explanations in the discussion (just a suggestion).

Response 8. We don’t think that roadkill data is inherently bad but we recognize that there are many questions surrounding its use and interpretation of results. However, given that Koen et al. (2014) found a correlation between roadkill and current density in eastern Ontario, we indicated in our discussion that the most likely explanation for difference in results was the spatial resolution of our movement cost layer. It should be noted that we did not use point data for our analysis but rather used the mean current density for the focal pixel and the neighbouring 8 pixels to capture current densities adjacent to the roads. We agree that it would be interesting to perform additional analyses at multiple scales in order to explore home-range selection but we are saving that for a more comprehensive analysis in the future.

**9)** Results (lines 293-322, and Figs 1-2-3): Is this really the most important results of your paper? I know these are needed to understand the following, but these are only methodological stuff, i.e. intermediate steps to reach your goal that was to model connectivity for several species throughout Canada (get back to your manuscript title… nothing about the methodological contributions, just a focus on a pan-Canadian connectivity modeling exercise…). Please considering sending all of it to an Appendix or Supplementary Material.

Response 9: Thank you for your advice, which we have followed in the revision and moved the figures to Supporting Information.

**10)** Results (lines 331-337 and Fig. 4): This is obvious, less resistance in the south, more in the north. As are the results regarding connectivity (less in the south, more in the north). Despite this, these two figures (4-5) are very interesting, and deserve publication. But I think that the message they brought could be presented in a more efficient way, i.e. via conservation issues and management imperatives. Please consider revisit the scope of your manuscript, with less methodological findings, you’ll have more room to describe the findings (locally, regionally, nationally) and potential outcomes of your work.

Response 10. Revised as suggested.

**11)** Results (validation – lines 398-406): Here I have some doubts regarding the use of GPS-telemetry relocations to validate the connectivity corridors (current density). Looking at lines 400-402, I see the number of data locations, but not the number of animals (as I suspect that you didn’t used 17,444 independent wolves!). So this validation exercise appears to me overly powerful (from a statistical perspective), as the pseudoreplication associated with the same animal having several relocations at one place doesn’t seem to be properly considered in the validation process. Were the relocations belonging to one given animal considered as replicates of this animal, i.e. (explained more clearly, I hope…) that the animal ID is used as a random factor (strata) in the analysis? Also, why using random points vs. GPS relocations? Based on the 95% kernel UD, several random points will end being overlapping real relocations, which would probably decrease the level of contrast between random and GPS points (see figure 8). Why not using 10 or 20 bins of current density to figure out how many relocations (or individuals) were seen in each of these strata of current densities? I am not sure this would be the best way to use your GPS data, but at least it seems to me that it could deserve some more attention given the issues raised above in my comment.

Response 11: We thank the reviewer very much for pointing this out, and we have revised our analysis of the independent movement data to include individual as a random effect (lines 260-275). We also clarified how many individuals versus how many GPS observations were used (lines 251-254). We used each species full GPS datasets to estimate home ranges and 10% of their data (randomly selected) to examine current density values and avoid the potential effects of spatial autocorrelation. We compared current density at observed locations to current density at available but unused locations (random points outside home range) as a modified application of the used/available design typical in resource selection models (e.g., Manly et al. 2002, Boyce 2006), for understanding habitat preferences. We used the 95% kernel UD to exclude areas with the very lowest probability of use and thus strengthen the contrast between what was used and what was available (we added this as clarification to lines 265-266). In this revised analysis, we used current density as the response variable, and case (i.e., observed location = 1; random location = 0) as the categorical predictor, with individual as a random effect. We also included an interaction between ‘case’ and individual’s max displacement distance to examine whether the selection for higher current density values was more important for individuals who moved further, compared to individuals who covered relatively smaller areas. We used the upper quartile bin (0.75-1.0) of maximum displacement distances to identify the long-distance movers and the lower quartile bin (0-0.25) of maximum displacement distances to identify the short-distance movers, and we provide this explanation in lines 277-289 and a histogram with binning of distances in S5 Fig. For all species the long-distance movers selected for higher current density values than what was available (Fig 6).

We also examined current density using the binning approach suggested by the reviewer, which we show below. We did not include this analysis in our manuscript because it can be misleading in areas of dispersed current flow (see the caribou panel below). Areas of dispersed flow, where most of the caribou GPS locations occurred, are areas where moderate current density values (i.e., middle quantile bins) indicate many pathways for movement. These places occur in areas that are largely intact, and thus good for connectivity, but do not have the highest current density values (which tend to occur where current is concentrated in between areas of higher cost to movement). See Barnette & Belote 2021 and Brennan et al. 2022 (references 20 & 26) for discussions regarding the importance of dispersed flow in the literature.

Bar plot depicting the number of GPS locations within five current density quantile bins. Highest current density values are within the 0.8-1.0 quantile bin.

**12)** Results (lines 402-404): unclear to me: 0.04 is significant, did I miss something?

Response 12: Thank you for catching that. We have corrected the number.

**13)** Discussion (general comment): The discussion also could benefit from deciding if the focus of this manuscript is more methodological or ecological. But my main point here is that an important part of the discussion is repeating what was done (methods, e.g. lines 423-431, 450-454, 476-517) or what was found, less on comparing these results (and their interpretation) with the published peer-reviewed literature. Please understand me correctly: I really enjoy your manuscript, but was disappointed to see that the discussion section was not discussing the results as deeply (in details) as what we could expect from this section.

- Were the corridors found confirming corridors found locally, regionally or provincially in other studies? Were they corresponding to interesting corridors already highlighted for other species than those you have used?
- Very few references are cited in this section, and I know (having read other papers published by the different coauthors) that you could build a stronger discussion.

Response 13. Thank you, we have revised the manuscript based on your suggestion and added content and references to the discussion. We were also interested in whether our results confirmed corridors that have been identified as part of connectivity conservation initiatives, but some of the better known initiatives are so broad and generalized (e.g., Y2Y, Staying Connected) that there was no meaningful way to statistically analyse for correlations. Instead we opted for highlighting a few initiatives and describing the concurrence with areas of high current density on our map. However, we fully intend to determine whether areas of high current density on our map correspond to corridors identified in species-specific studies, conditional upon the interest of other researchers, of course. We also hope to compare the results of our omnidirectional analysis to analyses that used the source to destination approach.

**14)** Discussion (lines 533-534): So what would be the minimum size of a species for which your tiles can be efficient at modeling connectivity? Or what would be the spatial resolution that you recommend to use for herpetofauna?

Response 14. We have clarified that the study by Koen et al. (2014) found a significant correlation between herpetofauna roadkill and current densities using a 100 m resolution cost surface. Lines 478-481

**15)** Discussion (lines 557 +): I really appreciate the potential limitation section.

Response 15: Thank you!

**16)** Conclusion (general comment): The conclusion is mostly repeating things that we have already read in other sections of the manuscript. Could you revisit this section to add some recommendations? Potential outcomes of the use of your connectivity map to contribute to the conservation of different species and wildlife habitats?

Response 16: Acknowledged! We have revised the discussion.

**17)** Figures 1-2-3: maybe it’s just the way the figures were joined to the manuscript, but they can be smaller, all presented into one figure with several panels.

Response 17: We have joined figures 1, 2, and 3 as suggested, but move them to the Supporting Information in response to comment #9 above.

**18)** Figure 9: Sorry but I cannot see the positive correlation here… the relationship appears to me negative. Have you tried to put a GAMM or a non-linear curve on this group of points? I am surprised that this relationship is significant; please explain more in details these opposite perspectives between your text (positive relationship) and your figure (negative relationship, although strongly driven by a few points).

Response 18: The correlation is difficult to see but we confirmed the results by repeating the analysis and conducting a Pearson correlation analysis with standardized data for further support. We have also altered the figure so the points are open circles, to help make more points visible.
