# Supplementary material for: Predicting areas important for ecological connectivity throughout Canada
Source: PLoS One. 2023 Feb 22;18(2):e0281980. doi: 10.1371/journal.pone.0281980 (PMC9946242; doi:10.1371/journal.pone.0281980)
Supplement: Attachment — Submitted filename: Response_to_Reviewers.docx [file pone.0281980.s010.docx]

PONE-D-22-17312R1

Predicting Areas Important for Ecological Connectivity Throughout Canada

**RESPONSE TO ACADEMIC EDITOR**

Thank you for the opportunity to further improve our manuscript. We have made more revisions in response to the reviewers very helpful comments, as outlined further below. We truly appreciate the time the reviewers have contributed to this process, resulting in what we believe is a much stronger manuscript.

We have been able to respond to all the review comments, which were mostly straightforward. We disagree with R2’s opinion regarding omnidirectional methods, which we tried to address in the first round of responses. This method is frequently used in the connectivity conservation literature (outlined below) as it helps to address the biases that can occur using source-to-destination connectivity methods, which we describe in lines 122 – 130 and 223 – 228 (**all line numbers correspond to those in “Revised_Manuscript_with_Track_Changes.doc” when viewed using ‘All Markup’ in Track Changes**). We also note that it is unrealistic to expect that all source and destination nodes would be known, especially for multiple species throughout Canada, and for the many different applications of connectivity analyses.

As mentioned before, the omnidirectional connectivity method was first published in PLOS One (Pelletier et al. 2014) as well as in Methods in Ecology and Evolution (Koen et al. 2014) and has since been used in numerous other studies (a combined 287 citations according to Google Scholar; with specific examples included in our response to the first review). Nevertheless, we indicate towards the end of the Discussion (lines 758 - 760) that it would be desirable to compare the results of our omnidirectional analysis to results from species-specific and park-to-park connectivity analyses. We have started to reach out to other researchers for more species’ data and model results and hope that their use in a future analysis will help address Reviewer 2’s skepticism regarding the value of the omnidirectional method.

With respect to R2’s concern about the use of expert opinion, we acknowledge that in general, it is less desirable than empirical data. It is important to appreciate that collecting movement data for many individuals of many species typically incurs massive financial costs and logistical challenges, which is why one of the stated objectives of our research was to determine whether a simpler approach could be used to model multi-species connectivity across Canada. Therefore, using expert opinion was an explicit goal of our study. As we describe in the manuscript, we used only four movement cost values (more would lead to greater uncertainty in the results) and the values we assigned were consistent with other studies (references: 6, 15, 17–20, 22, 26, 31–34) and based on published studies of species’ biology, which we cite (6, 34, 39, 40-42, 45-49). Most importantly, our resulting current density map was validated with independent wildlife data.

Although we understand R2’s perspective on structural versus functional connectivity, we also agree with the reviewer that these approaches exist on a gradient (see Reviewer 2’s comment that “your approach is closer to a structural view of connectivity than a functional one”). As R3 also noted in the Round 1 comments, connectivity models are usually on a “gradient” between the simplified dichotomous concepts of structural and functional connectivity. The very act of parameterizing a movement cost layer based on generalized species movement patterns adds some degree of functionality. Ultimately, however, the fact that independent wildlife movement data was found significantly more often in areas with high current densities compared to available but unused areas demonstrates that our model was indeed predicting areas of functional connectivity. We were intrigued by R3’s point (round 1) that it would be very interesting to further discuss the structural - functional gradient, but we feel that it merits its own manuscript.

We have provided responses to each reviewer comment below, and we look forward to your further review.

Two references related to the CBD Post-2020 Biodiversity Framework were replaced with a single reference to support changes in the text, which now refers to the adoption of the Framework in December.

Thank you very much,

Richard Pither

**====================================================================**

**Reviewer #1**: Thank you very much for your attention to detail in addressing the comments from all three reviewers in this revised version of your manuscript. I have paid particular attention to the responses to my original comments and am happy with all responses you have provided.

As for the other reviewer’s comments, I have read over them as well and even though I might not agree with some of the points the reviewers made I do appreciate the attention and care you have used to address each and every comment.

I have no further comments at this point and am happy to recommend publication of this manuscript.

Best regards,

Richard Schuster

Director of Spatial Planning and Innovation, Nature Conservancy of Canada

Email: richard.schuster@natureconservancy.ca

**====================================================================**

**Reviewer #2**: I thank the authors for their responses to my comments. I have some follow-up comments. And I apologise for not being more supportive, but I feel that my biggest concerns were not adequately addressed, or perhaps not fully understood. There are three major issues: (1) the use of expert opinion; (2) related to this, the authors insistence that relative costs do not matter, when there is evidence to show that they do; and (3) use of omni-directional approaches that ignore 'nodes'.

I'd like to say that I appreciate the scale of the landscape, and that there can be substantial practical application for such an effort, with government policy backing. However, this makes it all the more important to have good science, and good connectivity maps that are based on ecological knowledge and empirical data.

I have provided my detailed comments below but before that, I can provide some pointers on how to address these:

1. Use of **expert opinion**: State from the beginning that the authors take a structural approach to connectivity (different from an upstream approach); and that the authors expect such an approach to be useful for a large number of species that are dependent on natural ecosystems / specialists to natural ecosystems. Clearly state (even in the abstract) that the cost was based on expert opinion, while the movement model results were validated with empirical data on such species (i.e., clarify that empirical data was only used for validation).

**RESPONSE 1**. We sincerely appreciate the time the reviewer has taken to review our manuscript. We have addressed several comments in both rounds of reviews and are confident the manuscript has improved as a result. For responses to the pointers, please see details below. For this specific pointer, please see detailed Response 4.

2. Relative costs vs ranks: Clearly state throughout the MS that relative ranks matter (ie., what has been referred to as range of values). Run the sensitivity test against high-movement / pinch-point areas alone (e.g., correlation of top 10% movement areas -- or areas which would be identified as corridors, or overlap of identified high-priority connectivity areas across scenarios). Include this as a caveat to your manuscript.

**RESPONSE 2**. Please see detailed Responses 10 - 13.

3. Omnidirectional connectivity: Have a clear explanation for what this means. As of now, you have provided corridors identified for a hypothetical scenario where all animals are at the boundary of Canada. Please state -- what would this signify for the species ecologically and/or from a conservation perspective. Include a caveat that if data on animal distribution were available, it would be an improvement to add such information.

**RESPONSE 3**. Please see detailed Responses 14 - 17.

Detailed comments:

1. Use of expert opinion:

Response 1- I accept that there can be two approaches, one where connectivity is modelled for each species and then combined, and one where the 'resistance' is combined across all species. My point was not this. My point was that you use expert opinion to parameterise (multi-species) resistance. Expert opinion is problematic for accurately reflecting the resistance of a single species; it is going to be as, or much more, difficult to reflect the needs of multiple species.

You note in lines 142-143 that: "Cost surfaces can be constructed using expert opinion or empirical data". This is true, but, there has been a move away from expert opinion and specific calls for such a move.

**RESPONSE 4**: Although we agree that empirical data has obvious advantages over expert opinion, one of the objectives of our study, as stated in the Introduction, was to determine whether a simpler approach could be used to model multi-species connectivity across Canada. We have updated the abstract (line 29) and the last paragraph in the Introduction to explicitly state our use of expert opinion (line 138).

**Note: all line numbers correspond to those in “Revised_Manuscript_with_Track_Changes.doc” when viewed using ‘All Markup’ in Track Changes**.

—---------------------------

With this -- and because your expert opinion was based on how far a location is from 'natural-ness', I would suggest that your approach is closer to a structural view of connectivity than a functional one. There is no issue with using a structural view, but your manuscript needs to accurately and truthfully reflect this. You can clearly say that you have used a structural approach - validated with locations of multiple species - that assumes that this will be relevant for a set of species that are dependent on natural environments.

**RESPONSE 5**: As implied by the reviewer, connectivity models can fall on a gradient between the structural and functional connectivity. The steps of parameterizing a movement cost layer based on generalized species movement patterns and using a random walk algorithm to model individual movement adds some degree of functionality. More importantly, our resulting current density map was validated with independent data for several wildlife species. Specifically, GPS telemetry data from individuals that moved longer distances (wolves, caribou, moose, elk) were found significantly more often in locations of high current densities than unused but available locations. Therefore, we consider that our map predicts functional connectivity for select species. We recognize however, that the gradient exists between these model types and have tried to make that clear in the discussion. We removed the word functional from the first sentence of the discussion and have added a new paragraph where we briefly address these concepts (Lines 580 - 586):

- “Use of an upstream, generic species approach[9] and a human footprint could be considered a model of structural connectivity. However, we attempted to include elements of functional connectivity by ranking our movement costs based on published studies of species’ biology and employing a random walk algorithm to model movement. Furthermore, the predictions of our model were supported by independent movement data for some species. Indeed, we consider that there is a gradient between the concepts of structural and functional connectivity, and that our study has aspects of both types”

—----------------------------------------------------

Note: A structural approach cannot be interchangeably used with an upstream approach, in its absolute sense. An upstream approach speaks to collating data across species at the 'resistance' parameterisation step. You assume that the expert opinion approximates these data. Thus, even in an upstream approach, empirical data has a place, has value, and your work replaces that with expert opinion. This is fine (not ideal), but you need to be very clear about it. And you do have to discuss this as a caveat in the discussion.

**RESPONSE 6**: We agree that those terms are not interchangeable and have not used them as such. As we noted in the manuscript, we were following the Wood et al. (2022) definition, which states : “Upstream methods, such as species agnostic or **generic species approaches**, require substantially less species-specific data and computational resources” (our emphasis with bold text). We described this generic species approach in lines 85 - 99. We have now revised our stated objective in the Introduction to clarify that we used expert opinion (line 138). Our abstract already stated that we “... developed a movement cost layer with cost values assigned to anthropogenic land cover features and natural features based on their known and assumed effects on the movement of terrestrial, non-volant fauna.” Importantly, our resulting current density map was validated with independent data for several wildlife species.

—------------------------------------------------------

Also, Response 4 & 5: Thank you for clarifying the upstream and downstream approaches. Throughout, I'd like to clarify that this bit of your approach is now clear to me. What I (was and) am interested in is: within your upstream approach, how you incorporated species-movement information or empirical data. When combining species connectivity maps, there are multiple approaches: you could take the locations that matter to the most number of species; you could sum current (ie., choose locations that contribute the most across species); you could choose pinch points for a set of species. What would be your associated approach here. Are you seeing this as taking least-common-denominator of species requirements for movement (natural-ness), or do you see this as encompassing the maximum number of species, or do you see this as encompassing there most sensitive species?

**RESPONSE 7:** Yes, we essentially used the common-denominator of species’ movement behaviour in response to anthropogenic and natural land cover features. We stated that “we modelled connectivity for terrestrial, non-volant fauna that use and move across natural land cover more successfully than anthropogenic land cover types by assigning higher costs to landscape elements with higher degrees of anthropogenic disturbance.” (Line 158 - 160). Meaning we tried to encompass the maximum number of species that are sensitive to anthropogenic disturbance. Although we did not use animal movement data to model and parameterize the movement cost layer, we constructed the layer and assigned cost values consistent with other studies (references: 6, 15, 17–20, 22, 26, 31–34) and based on published studies of species’ biology, which we cite (6, 34, 39, 40-42, 45-49) in a few locations, including lines 148-149. And we did so to see if such an approach would predict areas important for connectivity that would correspond with observed animal movement patterns. This is explained in lines 75 - 85 and 539 - 541. Furthermore, we explicitly state that our analysis was designed to predict connectivity for species that “move across natural land cover more successfully than anthropogenic land cover types” (lines 207 - 211). Importantly, our approach was validated using independent wildlife data, which indicates support for our approach.

—-------------------------------------------------------

Side note: I'm a bit confused about your statement: "there is no common set of species that occur across all of Canada". Of course there won't be, but there doesn't have to be. A corridor will serve more species, where there are more species; and it will not be useful in a landscape with (as a caricature example) there are no species. It is meaningless to have a jaguar corridor in Montana -- again a caricature example - or even in Northern California. All of this makes my worry about the practical applications of this output. Assuming that this will go into some sort of corridor demarcation or policy, how will it account for the lack of the realism in terms of not reflecting underlying species range limits and distributions; in terms of not specifying what is being connected where.

**RESPONSE 8:** The manuscript states that the goal of this project was to test the upstream approach using a generalized movement cost layer, with costs assigned to reflect those for species whose movement patterns are known to be negatively affected by anthropogenic features. We also state that the “upstream approach we used undoubtedly does not capture the connectivity needs for all species, nor all complex movement patterns or population processes …” (Lines 756 - 758). We agree with Reviewer 2’s implication that our current density layer alone should not be used to demarcate corridors. Indeed, our current density layer is being used already *in combination with other data layers and local knowledge* by governments and agencies to prioritize conservation decisions and avoid the identification of meaningless corridors. But we consider it useful to have a seamless map produced in a consistent way across an area as large and diverse as Canada, where a common currency (current density) can be considered among other values.

—----------------------------------------------------------------------------

From the author response: "This is important in the context of providing advice for applied land-use planning, where details about node placement may vary from application to application." Could you please explain why node placement would vary? Do you mean from species to species?

**RESPONSE 9:** We meant that because land-use planning involves multiple objectives and species, node placement for different objectives would most likely vary. E.g., Different nodes would be required when identifying areas important for connectivity between two specific protected areas, identifying locations for wildlife passages to reduce road mortality in areas with no protected areas, identifying potential corridors between disjunct populations for an endangered species, or for recolonization of habitats following local extinctions. We also reiterate that in many situations, sources and destinations are not known, especially for multiple species throughout much of Canada (lines 131 - 133).

—--------------------------------------------------------------------

2. Relative cost rankings:

Response 12: The absolute cost values do not matter. But the relative cost, in addition to the rank order of matrix types matters and this has been shown previously (i.e., ratio vs ordinal data).

Rayfield et al. (2010) found that: "spatial location of least-cost routes was sensitive to differences in relative cost values assigned to landcover types". From Bowman et al. (2020): "We also found that correlations were more variable when the range of cost values in a map was high". Also Fig. 3.

Which means that statements such as this the one below (lines 168-170) made in the MS are misleading and patently wrong. (lines 168-170: "Current density maps have low sensitivity to the absolute value of the costs assigned to land cover types so long as the relative ranking of the types is maintained" Not only do you need to maintain the relative ranks, you also need to retain the ratio of costs across categories. This has been shown by both citations, see above.

**RESPONSE 10**. We think there is some confusion about terms here. It appears that the reviewer uses the phrase “relative costs” to mean much the same thing as the ratio of the costs, whereas in the statement at lines 183 - 185 that “Current density maps have low sensitivity to the absolute value of the costs assigned to land cover types so long as the relative ranking of the types is maintained." We use relative to indicate rank order of costs relative to one another. To clarify this, we have now removed the word relative and rephrased this sentence as “Current density maps have low sensitivity to the absolute value of the costs assigned to land cover types so long as the rank of the types is maintained". This was the conclusion of the paper by Bowman et al. (2020) after a simulation study (the lead author of that paper is an author of the current research being reviewed here). There is a small effect of the range of costs and of the ratio of the costs, but the rank order of the costs is overwhelmingly the most important effect. Therefore, sensitivity to these other effects is relatively lower (i.e. low sensitivity). This is the simple, important point we are trying to make. The mean correlation in current density values across all scenarios with different relative cost values but maintaining rank order in the Bowman et al. (2020) paper varied from 0.87 to 0.90 depending on fragmentation level. Therefore, Bowman et al. (2020) concluded sensitivity to absolute costs was low. We also note that the Rayfield et al. (2010) paper is a bit less relevant to the questions here because it deals with sensitivity of least cost paths which have different properties due to the route optimization.

—--------------------------------

The authors themselves go on to say: (lines 172-174) "However, the range of cost values (e.g., 1 to 3 versus 1 to 1000) does appear to have a small effect on the pattern of current densities". This is the effect of the relative values of costs, and it is not a small effect.

**RESPONSE 11**. Please see Response 10. The mean correlation in current density values across all scenarios with different relative cost values but maintaining rank order in the Bowman et al. (2020) paper varied from 0.87 to 0.90 depending on fragmentation level. We consider the remaining unexplained variation to be a small effect. Perhaps this is semantics, but it is at least a much smaller effect than the variation due to the rank order of the costs.

—--------------------------------------

And, they agree with this in responses 31 and 32. Yet, it is not reflected in the methods section, and methodological approach of the MS.

**RESPONSE 12.** We explicitly address this variation due to different cost scenarios using a sensitivity analysis. See S2 Table and S2 Figure for details. This is the very purpose of the sensitivity analysis, which found that mean correlations across 10 different cost scenarios with the same rank order varied between 0.79 and 0.84 depending on location. Unexplained variation was due to relative differences in the costs. We selected a highly correlated scenario as the basis for our model.

—--------------------------------------

The authors say they don't understand what I meant with this statement: "the locations of high-movement in the cited paper across different relative cost categories changes" There are two implications in this statement:

a. Relative costs matter (not just ranks)

b. It is not the average current densities that are of most importance, ecologically speaking, it is the location of high-current densities. The location of high-current densities, and the intensity of current in these locations, changed when Bowman et al. (2020) changed relative ranks (range of ranks, as per the article), and in Rayfield et al. (2010).

But there is one additional implication, for response 23(b). Locations of high-movement are significantly more important, but proportionally of a smaller area in the landscape. If you do a rank correlation, you will not (a) detect if there is significant difference between high and low movement areas -- a big impact of changing relative ranks; and (b) your results will be overwhelmed by med-low movement areas that are proportionally more represented in the landscape (ie., there is a large area covered by low-med movement, and a small area covered by high-movement). Hence, my reflection that this comparison is superficial, and will mask differences across your cost scenarios.

**RESPONSE 13:** The reviewer made this same point in the previous review and suggested that we scale current density values prior to correlating them to address the perceived problem. We did this in response to the reviewers comment last time.

From our response during the last review: Response 23. We prefer to use Spearman correlations in the manuscript because they are more robust than Pearson correlations to potential violations of spatial non-independence among the samples. However, we have also followed the reviewer’s suggestion here, and compared scenarios using mean-centred data (z scores) with Pearson correlations, which also demonstrated high correlations across all scenarios, supporting the conclusions we reached with the Spearman correlations (Maritimes: Spearman, mean r_s_ = 0.79, range = 0.39-1.0; Pearson of standardized values, mean r = 0.70, range = 0.25-1.0; BC: Spearman, r_s_ = 0.87, 0.63-1.0; Pearson, mean r = 0.79, 0.58-1.0). Therefore, after following the reviewer’s suggestion, we remain confident that current density tends to be highly correlated across cost scenarios provided that the rank order of the costs is maintained.

We remain satisfied that our comparison is not superficial, and that in fact, current density values tend to be highly correlated across scenarios provided that the rank order of the costs has not changed.

—-------------------------------------

3. Omni directional connectivity:

Response 2: The authors say that modelled connectivity patterns are dependent on node placement. But shouldn't it be? This is reflective of a reality wherein 'true' connectivity patterns will depend on where animals are present. If they are absent from an area, there will be no connectivity to that area. Note: This may or may not overlap with where Protected areas are; that does not determine the feasibility or applicability of this approach.

**RESPONSE 14**: We agree that there are situations where source and destinations nodes are known and as such should be specified in a connectivity analysis. For example, if the objective is to identify connections between a specific large wetland, which could be a source of frogs, and nearby ponds that could be potential destinations. But we were attempting to predict areas important for connectivity for multiple species throughout Canada. There are very few species for which all the sources and destinations are known, never mind multiple species. In addition, many species have continuous or semi-continuous populations in Canada, where sources and destinations lack meaning, whereas others species may have ranges that are spatially and/or temporally dynamic. We are also interested in identifying potential areas of connectivity for species that may not, in fact, be present at the moment. This can be important for planning for future connectivity (e.g., due to environmental change) or in anticipation of species recovery. We consider that the omnidirectional method was the appropriate method given our objectives, as others have in many other peer-reviewed studies. For example:

o	Hohbein RR, Nibbelink NP. Omnidirectional connectivity for the Andean bear (Tremarctos ornatus) across the Colombian Andes. Landscape Ecology. 2021 Nov;36(11):3169-85.

o	Choe H, Thorne JH. Omnidirectional connectivity of urban open spaces provides context for local government redevelopment plans. Landscape and Ecological Engineering. 2019 Jul;15(3):245-51.

o	Gray ME, Dickson BG, Nussear KE, Esque TC, Chang T. A range‐wide model of contemporary, omnidirectional connectivity for the threatened Mojave desert tortoise. Ecosphere. 2019 Sep;10(9):e02847.

o	McClure ML, Dickson BG, Nicholson KL. Modeling connectivity to identify current and future anthropogenic barriers to movement of large carnivores: a case study in the American Southwest. Ecology and evolution. 2017 Jun;7(11):3762-72.

o	Fleishman E, Anderson J, Dickson BG. Single-species and multiple-species connectivity models for large mammals on the Navajo nation. Western North American Naturalist. 2017 Jul;77(2):237-51.

o	Borja-Martínez G, Tapia-Flores D, Shafer A, Vázquez-Domínguez E. Highland forest’s environmental complexity drives landscape genomics and connectivity of the rodent Peromyscus melanotis. Landscape Ecology. 2022 Mar 15:1-9.

—-----------------------------------------

From your response: "current density patterns are biased by the placement of nodes (i.e., the same landscape will produce widely differing outputs depending on node placement)". As they should - if they weren't biased to node placement (reflecting our assumptions of where animals are), there would be a problem with our model. In that, I do not understand what movement, not dependent on node placement, means or ecologically signifies (because I cannot find the analogue for it in 'true' ecological patterns). Therefore, I do not know what the model will produce, and I do not know what to equate the model output with in terms of ecological processes. The authors need to clarify this, but have not.

**RESPONSE 15**: The model produces an estimate of current density, which is proportional to the probability of a random walk between all possible pairs of nodes, given the set of movement costs. We have added text in the methods to make certain this is clear (lines 182 - 183). Please see also Response 14.

—---------------------------

In response 28, the authors say: "a detailed exploration of the theoretical underpinnings of the method are beyond the scope of our current paper" and I agree, but the authors will need to at least be clear on, and state, what their results mean ecologically.

**RESPONSE 16**: Our model provides a seamless estimate of the probability of movement during a random walk for a set of terrestrial species that use natural cover across Canada. Further research and analysis will allow us to continue to define the set of species and conditions that are well depicted by the model, but for now we have validated the model with several species in different regions of the country. We have made sure this is clearly described in the introduction at lines 137 – 146, and in the methods at lines 180 – 183 and 267 – 269. Also please see responses 14 and 15.

—-------------------------------

I'd also point out that omnidirectional connectivity does not remove assumptions on node placement; they just move the nodes. Ie., you are assuming that animals want to move from the buffer of the map (where nodes are placed).

**RESPONSE 17**: Yes, we agree and this is exactly the point of the method. Nodes are moved to the buffer so that the resulting current density estimates inside of the modelled area of interest does not depend on node placement.

**====================================================================**

**Reviewer #3:** Good morning everyone,

I want to warmly thank the reviewers for their open-mindedness relative to the comments we all raised after reading the original version of their manuscript. I (Reviewer #3 on the first round) am satisfied with most of the changes made and the way the manuscript has been revisited. This is clearer now, more straightforward, less balancing between an ecological story and a methodological paper; I really think that the manuscript is stronger this way.

Nevertheless, I still have some minor concerns that may deserve some attention before going forward in the publication process. I have listed them below, hoping that they could be interesting for the authors in the finalization of their revised manuscript, and for the Editorial board to take a decision regarding this promising paper.

1) General comment #1 – structure (mainly discussion): Excellent revision of the structure of your manuscript, congratulations! The flow of arguments is clearer now, thank you. Nevertheless, I suggest adding another subtitle in the discussion, ~line 487, as the paragraph here (lines 487-511, maybe lines 487-529) seem to belong to another “section” compared to lines 432-485). While the lines 487-511 and 524-529 refer to the location of the corridors, lines 513-522 could be moved to the limitations or even more interestingly to the methods. In contrast, lines 433-485 explain more in details the strength of the exercise and the validation process.

**RESPONSE 18:** Thank you very much for taking the time to provide such helpful suggestions, which we feel have made the manuscript much better. We have inserted a section break as suggested (line 658).

**Note: all line numbers correspond to those in “Revised_Manuscript_with_Track_Changes.doc” when viewed using ‘All Markup’ in Track Changes**.

—----------------------------------------

2) Methods (lines 260-262): I suggest adding a few references in support to this decision, as the 10% of the GPS data sample for each individual seems quite a random decision and a random threshold.

**Response 19.** Thank you for this comment. We added to lines 342 - 344 that we randomly selected 10% of the data “as this is a common testing ratio used in habitat model validations (e.g., Heinemeyer et al. 2019) and can reduce the effects of autocorrelation”. See figures below for comparisons of model parameters estimated using different testing ratios). We tested three different testing ratios (100%, 10%, and 5%) and they show similar mean coefficient values and statistical significance across them.

Comparison of coefficient estimates for the species-specific linear mixed effects models (without interaction term) fitted to three different testing ratios of the GPS telemetry data: 100%, 10% and 5% of each individual’s data. From top left to bottom right, panels depict coefficient estimates for grey wolf, mountain caribou, moose and Rocky Mountain elk, Coefficient values for “Observed” depict the mean difference in current density values between observed GPS locations and available, but unused locations.

Comparison of coefficient estimates for the species-specific linear mixed effects models (with interaction term) fitted to three different testing ratios of the GPS telemetry data: 100%, 10% and 5% of each individual’s data. From top left to bottom right, panels depict coefficient estimates for grey wolf, mountain caribou, moose and Rocky Mountain elk.

—----------------------------------------

3) Methods (lines 294-296 and 303-304): Again, please provide some support to the decision of using a 300-m radius buffer for the herpetofauna roadkill data, and a 900 x 900m windows along roads for the moose roadkill data. In a recent paper, we (Laliberté & St-Laurent 2020, Landscape and Urban Planning) have shown that the radius buffer could influence (slightly, but this is a real effect) the strength of the correlation during the validation process. I suspect that maybe testing different buffer sizes would have influenced, even very slightly, the validation strength.

**RESPONSE 20**: We have added text to line 408 to clarify that used a 300 m radius around point data for herpetofauna roadkill to identify which pixels to use when taking the mean current density. Whereas because moose roadkill was so abundant and widespread, we ‘pre’ divided roads into 900 m sections (text updated in lines 414 – 416).

We also now note in the potential limitations section of the Discussion (lines 748 - 753) the role of buffer size. We are interested in further exploring the role of scale when using road kill data to validate connectivity in a future project. For this study, however, we were interested in the local scale; i.e., connections proximate to the road.

—----------------------------------------

4) Results (lines 316-318): I am not convinced that this reason (“… because that value has been used in previous studies”) is sufficient (or appropriate). CircuitScape cannot deal with null values, that’s why we usually add “+1” to all resistance values.

**RESPONSE 21:** Thank you. The manuscript has been revised to reflect your point (line 430).

—---------------------------------------

5) Results (line399-401 and Fig. 7): I think this could be important to bring to the attention of the authors that even if a positive, significant correlation has been found between current density and moose roadkill, this relationship is quite weak (see the very low Pearson r on Fig. 7). Looking at the figure suggest that this relationship is very “noisy” with a lot of variation in the number of roadkills for the same current density. This would open the opportunity to discuss the reliability of the different validation metrics in your discussion (as we did in Laliberté & St-Laurent 2020), and to identify potential other factors that may mask this expected relationship.

**RESPONSE 22:** We have revised the Results to be clear it was a weak correlation for moose in New Brunswick (line 517) and it was already mentioned in the Discussion (line 641 - 643). We have also added some discussion regarding other potential factors influencing roadkill hotspots, with examples (lines 645 - 651) and contrast that data type with GPS data as a possible explanation for why different types of data performed differently for validation (lines 651 - 655)

—---------------------------------------

6) Discussion (lines 462-463): Along with my comment just above, here you have to spend a little bit more time explaining why the different validation metrics performed differently, especially the moose roadkills. I suggest to view your result critically even if the relationship follows what was expected.

**RESPONSE 23.** Please see response 22.

—---------------------------------------

7) Discussion (lines 483-485): please add some examples, supported by the literature, for these “other factors”.

**RESPONSE 24.** Added (Lines 645 - 651).

—---------------------------------------

8) Discussion (Limitations): I REALLY enjoyed your section about potential limitations, nice job. You might have to consider talk about the “vignetage” issue that may be at play in some maps (see for examples Fig. 4a-b-c), but this issue needs more explanation in the methods (that’s why you overlapped some tiles…), especially regarding official boundaries (e.g. USA vs. Canada).

**RESPONSE 25.** We have revised the text to better explain how we used tiles and buffers to address the effects of node placement on current density in the Methods section, lines 254 - 255 and 261 - 267 and updated the map to better display the tiles in S4 Fig. We also added some text in the Methodological Advances section of the Discussion that discusses the issue of the anomalies in two regions (lines 719 - 726).
